# Anteroposterior Limb Skeletal Patterning Requires the Bifunctional Action of SWI/SNF Chromatin Remodeling Complex in Hedgehog Pathway

**DOI:** 10.1371/journal.pgen.1005915

**Published:** 2016-03-09

**Authors:** Shin Jeon, Rho Hyun Seong

**Affiliations:** School of Biological Sciences, Institute of Molecular Biology and Genetics, Seoul National University, Seoul, Korea; University of Oxford, UNITED KINGDOM

## Abstract

Graded Sonic hedgehog (Shh) signaling governs vertebrate limb skeletal patterning along the anteroposterior (AP) axis by regulating the activity of bifunctional Gli transcriptional regulators. The genetic networks involved in this patterning are well defined, however, the epigenetic control of the process by chromatin remodelers remains unknown. Here, we report that the SWI/SNF chromatin remodeling complex is essential for Shh-driven limb AP patterning. Specific inactivation of *Srg3*/*mBaf155*, a core subunit of the remodeling complex, in developing limb buds hampered the transcriptional upregulation of Shh/Gli target genes, including the Shh receptor *Ptch1* and its downstream effector *Gli1* in the posterior limb bud. In addition, *Srg3* deficiency induced ectopic activation of the Hedgehog (Hh) pathway in the anterior mesenchyme, resulting in loss of progressive asymmetry. These defects in the Hh pathway accompanied aberrant BMP activity and disruption of chondrogenic differentiation in zeugopod and autopod primordia. Notably, our data revealed that dual control of the Hh pathway by the SWI/SNF complex is essential for spatiotemporal transcriptional regulation of the BMP antagonist *Gremlin1*, which affects the onset of chondrogenesis. This study uncovers the bifunctional role of the SWI/SNF complex in the Hh pathway to determine the fate of AP skeletal progenitors.

## Author Summary

Anteroposterior (AP) limb skeletal patterning is directed by morphogen Sonic hedgehog (Shh) signaling. Modulation of Shh responsiveness and repression of Shh pathway activity in distinct limb bud regions are essential for proper limb skeletal formation. Although the genetic networks involved in these processes have been identified, epigenetic control by chromatin remodeler remains unknown. We have unraveled the function of the SWI/SNF chromatin remodeling complex in Shh signaling during limb patterning. The complex activates the responses of the posterior limb progenitors to Shh, however, it represses the signaling in the anterior limb progenitors. Here we provide genetic evidence for the dual requirement of the SWI/SNF complex in Shh signaling to pattern AP limb skeletal elements.

## Introduction

Vertebrate limb anteroposterior (AP) patterning is controlled by a diffusible morphogen, Sonic hedgehog (Shh), that is produced from the posteriorly located zone of polarizing activity (ZPA) [1]. Cell fate marking studies on mouse limb buds have revealed that Shh signaling regulates identities of limb skeletal elements, such as the ulna and digits 2 to 5, depending on the signal concentration and time of exposure to that signal [2–4]. During limb bud outgrowth, Shh promotes FGF signaling in the apical ectodermal ridge (AER) by mediating the BMP antagonist Gremlin1 (Grem1) that maintains low BMP activity [5].

In vertebrates, binding of Shh to its receptor Patched1 (Ptch1) enables the signal transduction through derepression of signal transducer Smoothened, allowing Gli transcription factors (Gli1−3) to function as activators (GliA) [6]. The transcriptional upregulation of *Ptch1* serves as a sensitive readout of Shh activity and is required for sequestering diffusible ligands to restrain their spread within the target range [7, 8]. Notably, the spatiotemporal regulation of *Ptch1* expression is important to prevent aberrant activation of Hedgehog (Hh) signaling, indicating that Ptch1 functions as a negative regulator of Hh signaling [9, 10]. Meanwhile, the full-length activators Gli2A and Gli3A contribute to the activation of Shh target genes such as *Gli1*, which might act as an indicator of the Shh signaling range in limb development [11–13]. The absence of Shh signaling allows proteolytic processing of bifunctional Gli2 and Gli3 to form the truncated repressors Gli2R and Gli3R (GliR) [14, 15].

Gli3 functions as a major regulator of AP digit patterning, whereas Gli2 has compensatory roles of Gli3 activity [4, 16–18]. During early limb bud development, *Gli3* is required to establish AP polarity through mutual antagonism with *Hand2* and is involved in the formation of two signaling centers, the ZPA and AER, by restraining GliA activity [10, 19–21]. In addition, constitutive *Gli3* expression during anterior digit patterning is mediated by repressing cell-cycle genes implicated in the proliferative expansion of Shh-dependent mesenchymal progenitors and by terminating *Grem1* expression to initiate chondrogenic differentiation [22, 23].

Despite recent progress in identifying networks of *trans*-acting regulators interacting with multiple *cis*-regulatory modules (CRM) that orchestrate limb development, epigenetic control of the developmental process, especially the role of chromatin remodelers, remains poorly understood. The mammalian SWI/SNF chromatin remodeling complex is an ATP-dependent chromatin remodeler that uses the energy of ATP hydrolysis to alter nucleosomal structure [24]. The SWI/SNF complex is a multisubunit complex including core factors such as ATPase *Brg1*, tumor suppressor *Snf5*, and scaffolding subunit *Srg3/mBaf155* (hereafter referred to as *Srg3*) [25]. In differentiation pathways, SWI/SNF complexes cooperate with histone-modifying factors and transcriptional regulators to mediate both transcriptional activation and repression in response to extracellular stimuli [26].

Here, we show that the SWI/SNF complex is essential for limb AP skeletal patterning. Specific inactivation of limb mesenchymal *Srg3*, resulting in defects in SWI/SNF complex activity [27], fails to upregulate posterior Shh/Gli target gene expression and induces the ectopic activation of target genes in the anterior limb bud after intact establishment of the ZPA. The SWI/SNF complex-mediated modulation of Shh responsiveness and repression of the ectopic Hh pathway determine the AP identities of limb progenitors and regulate the spatiotemporal expression of *Grem1*. Thus, bifunctional action of the SWI/SNF complex in the Hh pathway is essential to pattern AP limb skeletal elements.

## Results

### *Srg3* is essential for anteroposterior limb skeletal patterning

To study the specific function of the SWI/SNF complex in developing limb buds, we used a conditional loss-of-function allele of *Srg3* (*Srg3*^*f/f*^) [28] and a *Prx1Cre* transgene encoding a Cre recombinase that is activated in the early limb bud mesenchyme [29]. *Prx1Cre*-mediated inactivation of *Srg3* in the limb bud mesenchyme was confirmed by measuring the expression of the transcript and protein in control and *Srg3*^*f/f*^;*Prx1Cre* (hereafter shortened as *Srg3* CKO) limb buds. Whole-mount RNA *in situ* hybridization showed the specific clearance of *Srg3* transcripts throughout the mesenchyme and western blot analysis confirmed the downregulation of Srg3 proteins with a time lapse between the fore- and hindlimb buds (S1A and S1B Fig). In addition, the downregulation of Brg1 observed in *Srg3* CKO limb buds revealed the structural function of Srg3 that stabilizes the SWI/SNF complex (S1B Fig) [27].

**Fig 1:**
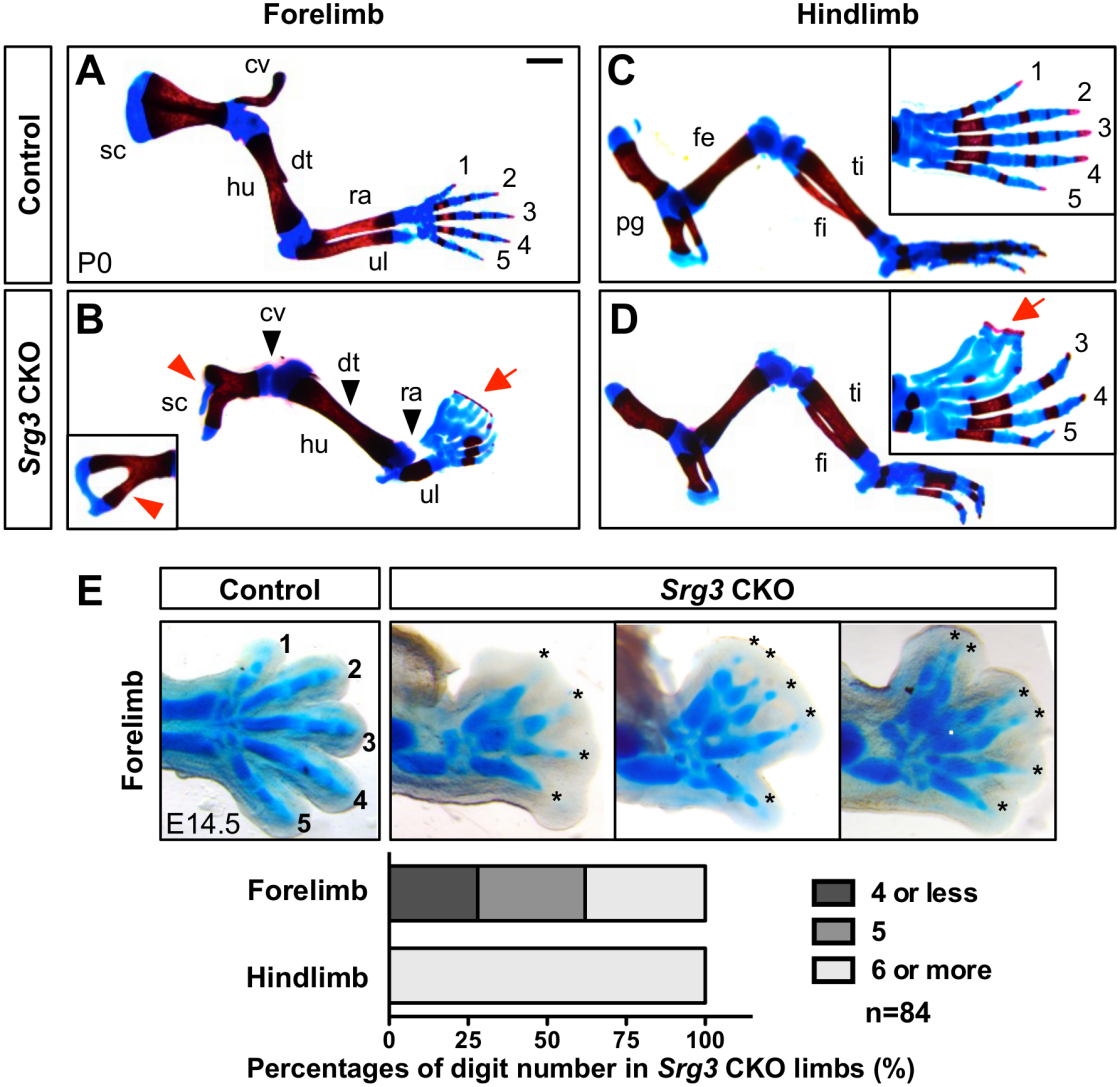
*Srg3* is essential for anteroposterior limb skeletal patterning. **(A−D)** Skeletal preparations of control and *Srg3* CKO limbs at P0. The inset in (B) shows another scapula phenotype. Red arrowheads denote hypoplastic scapulae and black arrowheads indicate the loss of clavicle, deltoid tuberosity, and radius in the *Srg3* CKO forelimb. The insets in (C) and (D) show a dorsal view of a hindlimb autopod marked with digit numbers. Red arrows point to the fused digits with soft tissues. cv, clavicle; dt, deltoid tuberosity; fe, femur; fi, fibula; hu, humerus; pg, pelvic girdle; r, radius; sc, scapula; ti, tibia; u, ulna; 1−5, digit identity. Scale bars: 1mm. **(E)** Percentages of digit number in *Srg3* CKO forelimbs and hindlimbs. Upper panels show various types of cartilage structures in *Srg3* CKO forelimb digits compared with control digits. Asterisks indicate hypoplastic digits.

Skeletal analysis of *Srg3* CKO limbs at birth (P0) revealed the requirement of *Srg3* for limb development (Fig 1). In *Srg3* CKO forelimbs, the scapula developed poorly with bifurcated or enlarged foramen, aplastic clavicle, stylopod (humerus) lacking deltoid tuberosity, and radial agenesis were observed (Fig 1A and 1B). In *Srg3* CKO hindlimbs, the proximal skeletons (pelvic girdle and femur) were retained normally, whereas zeugopod elements (tibia and fibula) were shortened to a similar extent (Fig 1C and 1D and S1C Fig). Both *Srg3* CKO fore- and hindlimbs had rudimentary digits that were connected by ossified tissues in the anterior digital tips (syndactyly) and exhibited more severe ossification defects in anterior digits than those in posterior digits (Fig 1B and 1D and S1D Fig). Unlike predominant preaxial polydactyly in *Srg3* CKO hindlimbs, digit number was variable in *Srg3* CKO forelimbs (4 or less, 28%; 5, 34%; 6 or more, 38%, n = 84) (Fig 1E). The discrepancy in severity between fore- and hindlimbs lacking *Srg3* is a likely consequence of *Srg3* deficiency mediated by the onset timing of *Prx1Cre* activity, which is first activated in the prospective forelimb bud prior to hindlimb budding [29]. Taken together, the malformation of zeugopod elements and variable digit numbers observed in *Srg3*-deficienct limbs suggest that mesenchymal *Srg3* is involved in AP limb skeletal patterning.

### *Srg3* CKO forelimb buds establish distinct Hh pathways in the anterior and posterior mesenchyme

**Fig 2:**
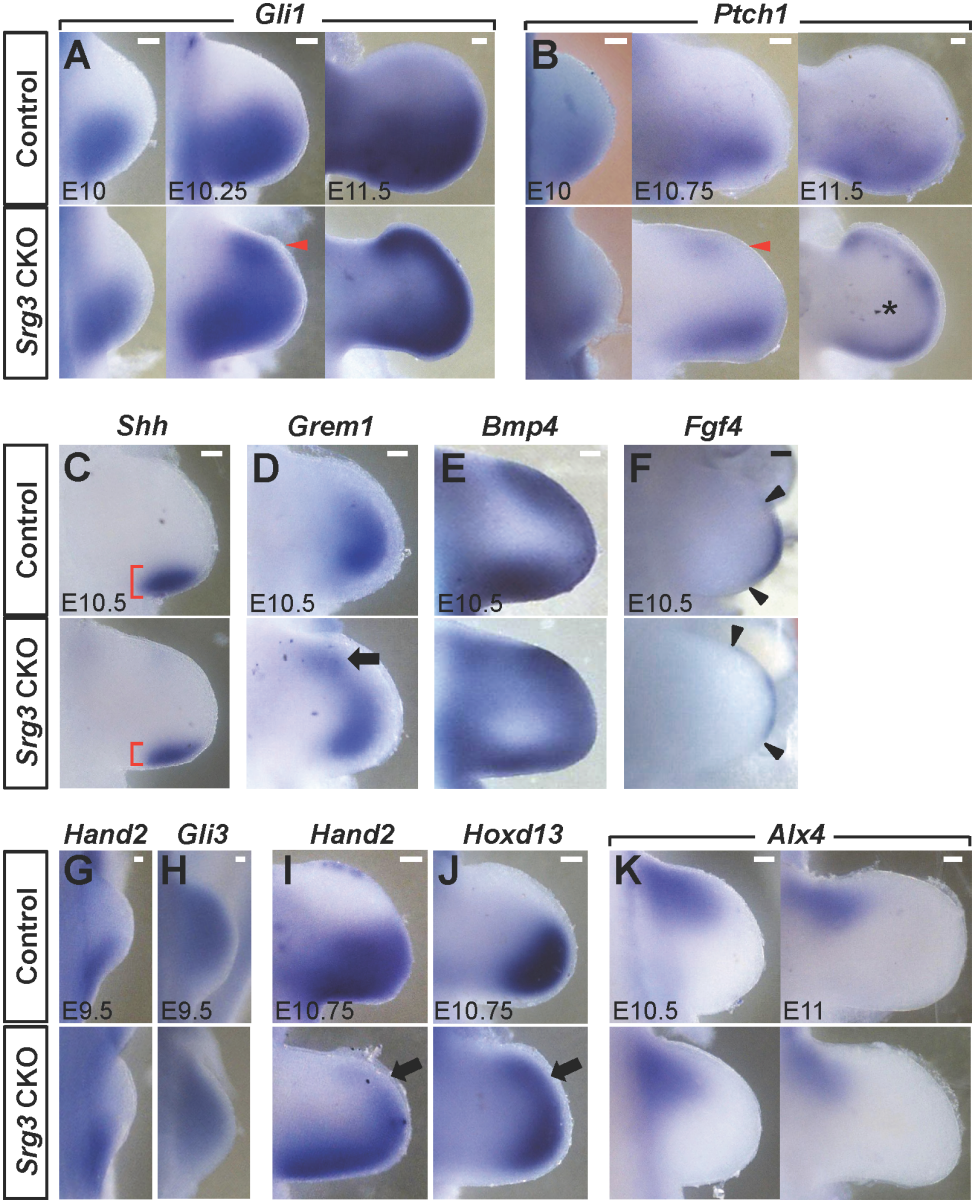
*Srg3* CKO forelimb buds establish distinct Hh pathways in the anterior and posterior mesenchyme. **(A, B)** Spatiotemporal distribution of *Gli1* and *Ptch1* transcripts in control and *Srg3* CKO forelimb buds at indicated stages. Arrowheads denote the ectopic activation of Shh target genes in the anterior mesenchyme. Scale bars: 100 μm. **(C−F)** Spatial distribution of *Shh*, *Grem1*, *Bmp4*, and *Fgf4* in E10.5 control and *Srg3* CKO forelimb buds. Brackets in (C) mark the spatial extent of the *Shh* expression domain. The black arrow in (D) indicates the anterior expansion of *Grem1* expression. Black arrowheads in (F) indicate the anterior and posterior end of the AER. Scale bars: 100 μm. **(G−K)** Spatial distribution of *Hand2*, *Gli3*, *Hoxd13*, and *Alx4* in control and *Srg3* CKO forelimb buds at indicated stages. Arrows in (I) and (J) indicate the anterior expansion of *Hand2* and *Hoxd13*. Scale bars: 50 μm in (G−H); 100 μm in (I−K).

Given that limb bud development requires formation of the ZPA and AER [5], we first analyzed the formation of ZPA and AER signaling centers at early stages. In E10 *Srg3* CKO forelimb buds, ZPA-*Shh* expression levels was similar with control expression levels (n = 8 limb buds analyzed), whereas AER-*Fgf8* expression was slightly reduced in *Srg3* CKO forelimb buds relative to controls (n = 6) (S2A Fig). Although *Srg3* inactivation did not significantly alter the formation of signaling centers, subtle changes in the AER suggest that the SWI/SNF complex functions in initial limb development. To understand the mechanism underlying Srg3-mediated limb AP patterning controlled by the counteraction of Shh and Gli3 [16, 17], we examined the expression of Shh/Gli target genes. In *Srg3* CKO forelimb buds, the expression domains of *Gli1* and *Ptch1* were normal up to at least E10 (*Gli1*, n = 12; *Ptch1*, n = 8), but were ectopically activated at E10.25 and at E10.75, respectively, in the anterior mesenchyme (*Gli1* and *Ptch1*, n = 6) (Fig 2A and 2B). In addition, *Gli1* and *Ptch1* expression was activated in a graded manner along the AP axis in control forelimb buds, whereas their expression domains including ectopic regions were confined to the distal region in *Srg3* CKO forelimb buds over time (Fig 2A and 2B; *Gli1*, n = 5; *Ptch1*, n = 6). Importantly, *Ptch1* transcripts were not detected in the core mesenchyme of *Srg3* CKO forelimb buds (Fig 2B). *Gli1* was ectopically activated from around E11 in *Srg3* CKO hindlimb buds, but its expression was comparable to control hindlimb buds in the posterior region (S2B Fig). These data suggest that *Srg3* both activates and represses Shh/Gli target gene expression in distinct regions.

To define whether bifunctional action of Srg3 in the Hh pathway affects the interlinked signaling between the ZPA and the AER [30], we examined the expression pattern of epithelial-mesenchymal signaling genes during limb bud outgrowth. In *Srg3* CKO forelimb buds, the size of the *Shh* expression domain was subtly reduced (Fig 2C; n = 6). *Grem1* expression expanded anteriorly (n = 7), whereas *Bmp4* expression was reduced in the anterior and posterior mesenchyme of *Srg3* CKO forelimb buds (n = 4) (Fig 2D and 2E). AER-*Fgf4* expression shifted anteriorly in *Srg3* CKO forelimb buds (Fig 2F; n = 6). Taken together, these data suggest that distinct Hh pathways established by *Srg3* deficiency differentially impacted epithelial-mesenchymal signaling in the anterior and posterior mesenchyme.

The polarization of nascent limb mesenchyme and establishment of the ZPA are controlled by antagonistic interactions between *Hand2* and *Gli3* in the posterior and anterior regions, respectively [19–21]. To assess whether *Srg3* deficiency in the limb bud mesenchyme affects AP polarity at the prepatterning stage, we examined the expression domains of *Hand2* and *Gli3* at E9.5. Consistent with the formation of an intact ZPA up to at least E10 (S2A Fig), the expression domains of these positional markers remained comparable to controls in *Srg3* CKO forelimb buds (Fig 2G and 2H; *Hand2*, n = 8; *Gli3*, n = 9). During limb bud outgrowth, the distribution of posterior markers *Hand2* and *Hoxd13* was more posteriorly restricted or reduced in *Srg3* CKO forelimb buds than in control limb buds, whereas their expression was activated in the anterior region at E10.75 (Fig 2I and 2J; *Hand2*, n = 7; *Hoxd13*, n = 5). By contrast, the expression domains of anterior markers *Alx4* and *Pax9* exhibited progressive decreases in *Srg3* CKO forelimb buds (Fig 2K and S3A Figs; *Alx4*, n = 6; *Pax9*, n = 6) [31]. Consistently, the expression of anterior markers was mildly downregulated in *Srg3* CKO hindlimb buds (S3B Fig), suggesting that the loss of anterior identity in *Srg3*-deficient limb buds correlates with the timing of *Srg3* inactivation. Taken together, these data indicate that *Srg3* deficiency progressively decreased the AP identities of limb progenitors, leading to a disruption of asymmetry after early specification of the AP axis.

### Mesenchymal *Srg3* deficiency induces ectopic *Shh* expression and distalizes epithelial-mesenchymal signaling at late stages

**Fig 3:**
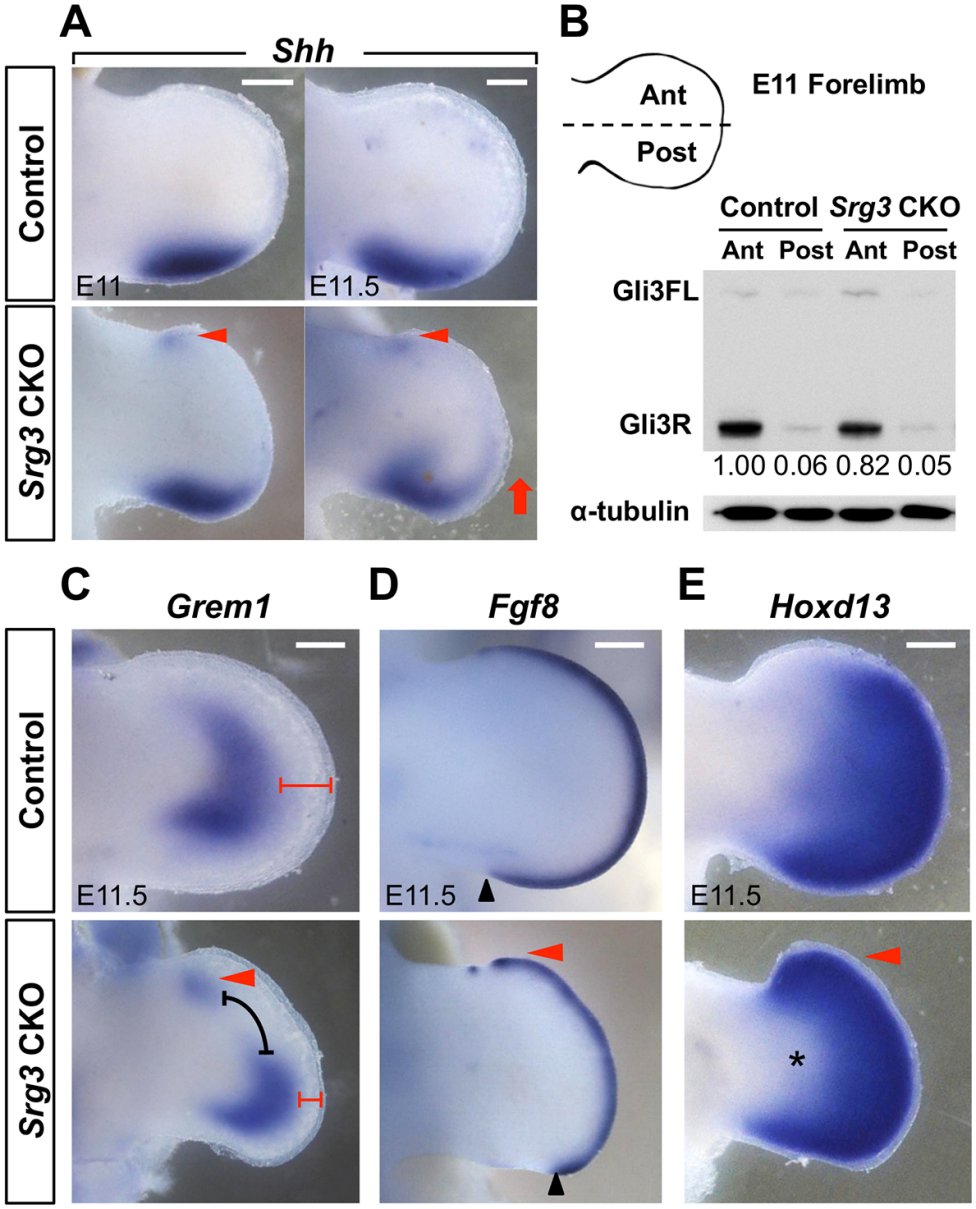
Mesenchymal *Srg3* deficiency induces ectopic Shh expression and distalizes epithelial-mesenchymal signaling at late stages. **(A)** Expression pattern of *Shh* in control and *Srg3* CKO forelimb buds at indicated stages. Arrowheads and arrow denote the anterior ectopic expression of *Shh* and its anterior expansion, respectively. **(B)** Western blot analysis of Gli3FL and Gli3R in lysates from the anterior (Ant) and posterior (Post) halves of E11 control and *Srg3* CKO forelimb buds. Values represent the relative levels of Gli3R protein. α-tubulin served as a loading control. **(C−E)** Spatial distribution of *Grem1*, *Fgf8*, and *Hoxd13* in control and *Srg3* CKO forelimb buds. Red arrowheads denote the anterior ectopic expression. In (C), the black bracket indicates the dissociation of *Grem1* expression domains and red brackets mark the distance between the distal expression of *Grem1* and the AER. Black arrowheads in (D) point to the posterior end of *Fgf8* expression. Asterisks in (E) indicate the distalized expression domain of *Hoxd13*. Scale bars in (A) and (C−E): 200 μm.

Inactivation of *Srg3* in the limb bud mesenchyme caused progressive alterations in Shh/Gli target gene expression and in AP identity (Fig 2). To gain further insights into the regulation of Shh/Gli target genes by the SWI/SNF complex, we reexamined the distribution of epithelial-mesenchymal signaling genes at subsequent stages. In *Srg3* CKO limb buds, *Shh* expression was ectopically induced in the anterior margin and subsequently expanded along the distal margin (Fig 3A and S4A Fig; n = 6 per stage). Ectopic Shh signaling reduced Gli3R protein levels by inhibiting Gli3 processing in the anterior mesenchyme of *Srg3* CKO forelimb buds (Fig 3B). To test whether the SWI/SNF complex is directly implicated in repressing *Shh* through the regulation of limb-specific *Shh* enhancer ZRS (ZPA regulatory sequence), which is responsible for localized expression of *Shh* [19, 32], we performed a chromatin immunoprecipitation (ChIP) assay. We did not observe the enrichment of Srg3 at any regions on the ZRS (S4B Fig). This suggests that ectopic *Shh* expression is indirectly induced in *Srg3* CKO limb buds.

After ectopic *Shh* expression was activated, the anteriorly expanded domain of *Grem1* at E10.5 was divided into two parts: the anterior domain and the posterior domain (Figs 2D and 3C; n = 7). In E11.5 *Srg3* CKO forelimb buds, the derepressed expression of *Grem1* in the anterior was remarkably reduced in the distal mesenchyme, whereas its posterior domain was distally shifted (Fig 3C). As the posterior domain of *Grem1* closer to the AER reflects loss of FGF signaling repressing *Grem1* [33], we assessed AER-*Fgf8* expression and found the thinning and posterior loss of AER together with ectopic upregulation in the anterior end (Fig 3D; n = 6). *Hoxd13* expression was also anteriorly expanded and confined to the distal mesenchyme in *Srg3* CKO forelimb buds (Fig 3E; n = 6). Likewise, the expression of *Grem1*, *Fgf8* and *Hoxd13* was ectopically upregulated in the anterior margin of *Srg3* CKO hindlimb buds (S4C–S4E Fig). Particularly, distalization of *Grem1* and *Hoxd13* expression domains was also observed in *Srg3* CKO hindlimb buds (S4C and S4E Fig). These data reveal that low Shh response and anterior Hh pathway activity by *Srg3* deficiency distalized epithelial-mesenchymal signaling and expanded the anterior digit progenitors.

### Srg3-containing SWI/SNF complexes are required for the transcriptional activation and repression of *Gli1* and *Ptch1* in developing limb buds

**Fig 4:**
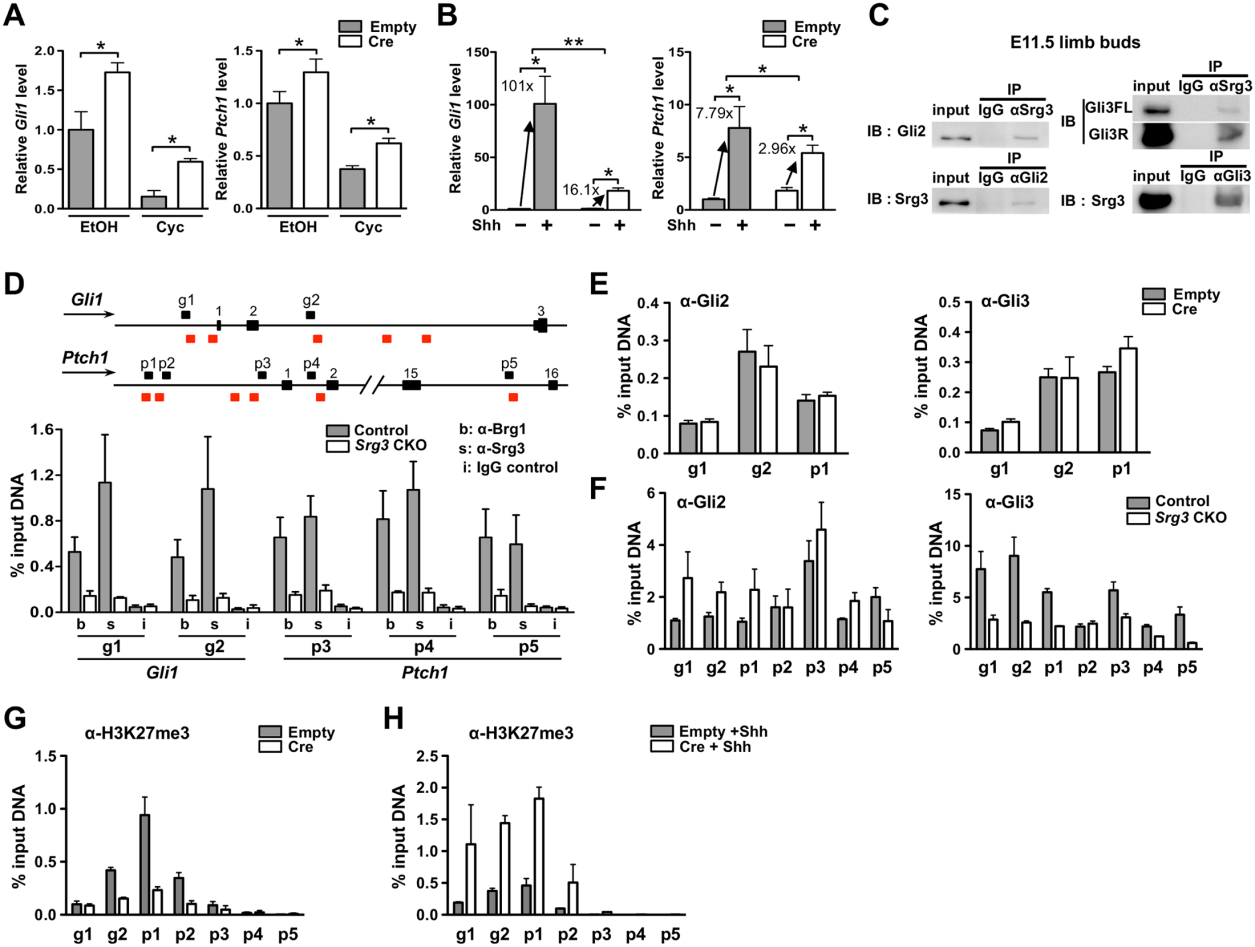
*Srg3* is required for the transcriptional activation and repression of *Gli1* and *Ptch1* in developing limbs. **(A, B)** Quantitative PCR analysis of *Gli1* and *Ptch1* mRNA in *Srg3*^*f/f*^ MEF infected with empty vector (Empty) or Cre-expressing viral vector (Cre). Each of infected MEFs was treated with an ethanol vehicle (EtOH) or cyclopamine (Cyc) (A), or incubated in Shh-conditioned media (+) or control media (−) (B). Error bars represent SD from six independent experiments. (*)*P* < 0.05; (**)*P* < 0.01. **(C)** Reciprocal immunoprecipitation of Srg3 with Gli2 and Gli3 proteins in E11.5 control limb buds. **(D)** Schematic representation of the relative positions of primer sets in the *Gli1* locus (g1, g2) and *Ptch1* locus (p1−p5) around the putative Gli-binding sites (red boxes) is shown on the top. Direction of transcription (arrows) and exons (numbers above boxes) are indicated. ChIP−qPCR analyses of DNA fragments precipitated with anti-Brg1 (b), anti-Srg3 (s), and IgG (i) in E11.5 control and *Srg3* CKO limb buds. (**E**, **F**) ChIP−qPCR analyses of DNA fragments precipitated with anti-Gli2 (left) or anti-Gli3 (right) in MEFs (E) and in E11.5 limb buds (F). (**G**, **H**) ChIP−qPCR analyses of DNA fragments precipitated with anti-H3K27me3 from MEFs cultured in a basal condition (G) or in Shh-conditioned media (H). All data in (D−H) are represented as the percentage of input DNA, normalized to the value of nonspecific binding to the *Gapdh* promoter. Error bars represent SD from three independent experiments.

To verify whether Srg3 directly regulates the expression of Shh/Gli target genes in developing limbs, we examined the effects of *Srg3* deficiency by transducing a Cre-expressing retroviral vector into *Srg3*^*f/f*^ mouse embryonic fibroblasts (MEFs). We focused our analyses on the transcriptional regulation of Shh/Gli target genes *Gli1* and *Ptch1*. Quantitative real-time PCR (qPCR) showed that *Srg3*-deficient MEFs expressed higher levels of *Gli1* and *Ptch1*, suggesting that the SWI/SNF complex represses Shh/Gli target genes (Fig 4A). To exclude the possibility that the SWI/SNF complex indirectly represses Shh/Gli target genes by other factors in the MEFs, we treated *Srg3*-deficient MEFs with the Hh pathway inhibitor cyclopamine [34]. Although cyclopamine reduced *Gli1* and *Ptch1* expression in control and *Srg3*-deficient MEFs, *Srg3*-deficient MEFs expressed higher levels of *Gli1* and *Ptch1* than control MEFs (Fig 4A). This indicates that the Srg3-containing SWI/SNF complex represses Shh/Gli target genes in, at least, a Hh-free condition. Thus, this finding could corroborate the derepression of Shh/Gli target genes in the anterior mesenchyme of *Srg3* CKO limb buds. Next, we examined whether *Srg3* is involved in the activation of *Gli1* and *Ptch1* expression upon Shh stimulation. In the presence of Shh-conditioned medium, *Srg3*-deficient MEFs displayed severely reduced activation levels of *Gli1* (101- vs. 16.1-fold) and *Ptch1* (7.79- vs. 2.96-fold), relative to controls (Fig 4B). These data suggest that *Srg3* is required for responses to Shh, supporting findings that the distribution of Shh/Gli target genes was confined to the distal-posterior mesenchyme in *Srg3* CKO forelimb buds.

During limb development, *Gli1* expression requires both transcription factors Gli2 and Gli3 [11], and Gli proteins regulate the expression of *Ptch1* [23, 35]. We asked whether the bifunctional action of Srg3 requires an interaction with Gli2 and Gli3 to regulate *Gli1* and *Ptch1* expression in developing limbs. Reciprocal coimmunoprecipitation of Srg3 with Gli2 and Gli3 from E11.5 limb bud lysates revealed that Srg3 formed a complex with endogenous Gli2, Gli3FL, and Gli3R (Fig 4C). Using previously reported Gli-binding sites [23], we assessed Brg1 and Srg3 occupancy at the regulatory regions of *Gli1* and *Ptch1* by performing chromatin immunoprecipitation followed by qPCR (ChIP−qPCR) in E11.5 limb bud extracts. ChIP−qPCR analysis showed that both Brg1 and Srg3 proteins were enriched at the promoter regions of *Gli1* and *Ptch1* around Gli-binding regions in control limb buds, whereas their occupancies were considerably diminished in *Srg3* CKO limb buds (Fig 4D). Furthermore, Brg1 and Srg3 were also enriched near the limb specific enhancer of *Ptch1*, which might be required for sensing graded Shh activity (Fig 4D, region p5) [35].

We next investigated whether loss of *Srg3* affects the recruitment of Gli2 and Gli3 proteins to the regulatory region of Shh target genes. The occupancy of Gli2 and Gli3 proteins was not significantly changed at the regulatory regions of *Gli1* and *Ptch1* in *Srg3*-deficient MEFs (Fig 4E). However, we found that Gli2 occupancy of Gli-binding sites was increased and the occupancy of Gli3 was reduced in E11.5 *Srg3* CKO limb buds at the promoter regions of *Gli1* and *Ptch1* relative to controls (Fig 4F, regions g1−g2 and p1−p4). These data indicate that Gli proteins bound to the Gli-binding sites were not affected by *Srg3* deficiency and suggest that their enrichment was differentially influenced by ectopic Shh activity in E11.5 *Srg3* CKO limb buds. By contrast, we also found the decreased occupancy of Gli2 and Gli3 proteins near the limb specific enhancer of *Ptch1* in E11.5 *Srg3* CKO limb buds (Fig 4F, region p5), suggesting that GliA contributed by ectopic Shh signals might have no significant effect on this region. We hypothesized that *Srg3* deficiency affects histone modification at the promoter regions of *Gli1* and *Ptch1* because the expression domains of *Gli1* and *Ptch1* were not expanded throughout *Srg3* CKO forelimb buds at E11.5, despite the high GliA and the low GliR condition. Indeed, SWI/SNF complexes functionally interact with histone modifying proteins [36–38]. Furthermore, Shh signaling induces a loss of a repressive mark, trimethylation of histone 3 at lysine 27 (H3K27me3), by switching histone modifiers from methyltransferase Ezh2 to demethylase Jmjd3 [39]. To test our hypothesis, we compared H3K27me3 enrichment at the promoter regions of *Gli1* and *Ptch1* upon *Srg3* deficiency against a previously reported distribution of H3K27me3 in MEFs [40]. Although Ezh2 and Suz12, components of the Polycomb repressive complex 2 (PRC2), were immunoprecipitated with both Gli2 and Gli3 in developing limbs (S5A Fig), there was no global change in H3K27me3 levels in *Srg3*-deficient MEFs or in the anterior and posterior mesenchyme of *Srg3* CKO limb buds (S5B and S5C Fig). At the enriched regions of H3K27me3 on *Gli1* and *Ptch1* promoters, however, *Srg3* deficiency resulted in decreased H3K27me3 level in a basal condition (Fig 4G, regions g1−g2 and p1−p2). Upon Shh stimulation, on the contrary, H3K27me3 levels at these regions in *Srg3*-deficient MEFs were significantly higher than in controls (Fig 4H). Taken together, these data suggest that Srg3-containing SWI/SNF complexes contribute to the activation and repression of Shh target genes through changes in the chromatin status of Gli binding regions.

### Loss of mesenchymal *Srg3* disrupted BMP signaling and caused defective chondrogenesis in forelimb buds

**Fig 5:**
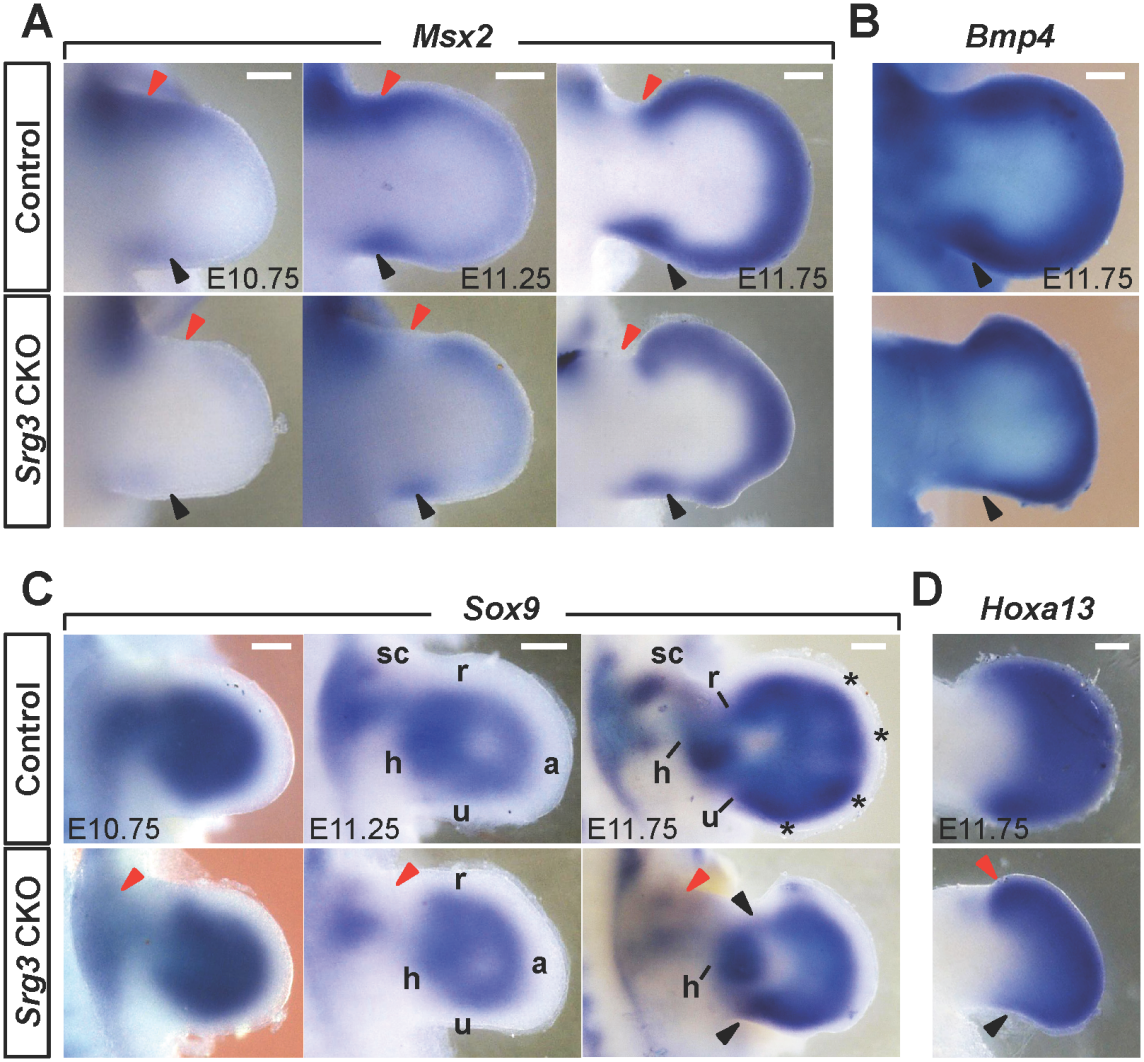
Loss of mesenchymal *Srg3* disrupted BMP signaling and caused defective chondrogenesis in forelimb buds. **(A, B)** Spatiotemporal distribution of *Msx2* and *Bmp4* expression domains in control and *Srg3* CKO forelimb buds at indicated stages. Red and black arrowheads indicate the anterior and posterior domains, respectively. **(C)** Spatiotemporal distribution of *Sox9* in control and *Srg3* CKO forelimb buds at indicated stages. Red and black arrowheads indicate the primordia corresponding to scapula and zeugopod, respectively. a, autopod; h, humerus; r, radius; sc, scapula; u, ulna; asterisks, digit rays. **(D)** Expression of *Hoxa13* in E11.75 control and *Srg3* CKO forelimb buds. Red and black arrowheads point to anterior expansion and posterior decrease, respectively. Scale bars in (A−D): 200 μm.

Posterior Shh signaling establishes limb skeletal structures including posterior zeugopod elements (ulna/fibula) and digits 2 to 5 [2, 4]. By contrast, loss of Gli3R or ectopic Shh signaling is detrimental to the formation of anterior skeletal structures [17, 31, 41]. To determine the role of bifunctional Srg3 in skeletal patterning, we assessed BMP activity, which promotes chondrogenesis at late stages [42]. In the anterior mesenchyme of *Srg3* CKO forelimb buds, *Msx2* expression, which marks BMP activity [30], was reduced at E10.75 and greatly abolished in the proximal region excluding the distal mesenchyme after ectopic *Shh* was induced (Fig 5A; n = 8 per stage). By contrast, posterior BMP activity remained low in *Srg3* CKO forelimb buds (Fig 5A). Among *Bmp* ligands, the expression of both *Bmp2* and *Bmp4*, but not *Bmp7*, was diminished in the posterior mesenchyme of *Srg3* CKO forelimb buds at E11.75 (Fig 5B and S6 Fig; n = 6 per gene). Concurrent downregulation of *Bmp2* and *Bmp4*, which are required to form the ulna and posterior digits 4 and 5 [43], could be causally implicated in the hypoplastic posterior skeletal elements of *Srg3* CKO forelimbs (Fig 1). These results indicate that *Srg3* deficiency disrupted BMP activities in the anterior and posterior mesenchyme.

We next examined the distribution of *Sox9*, which marks the condensation of chondrogenic progenitors [44]. Consistent with partially developed proximal skeletal elements, *Sox9* expression was diminished in the stylopod primordia of *Srg3* CKO forelimb buds prior to E10.75 (Fig 5C, left panel; n = 6). The expression of *Hoxa9*, *Hoxd9*, and *Hoxd10* of paralogous hox groups *Hox9* and *Hox10*, which control the formation of proximal skeletal elements [45, 46], was reduced in the proximal region of *Srg3* CKO forelimb buds (S7A Fig; n = 4 per gene). The expression of *Irx3* and *Irx5*, which are essential for patterning proximal and anterior skeletal structures [31], was also downregulated in the proximal anterior region of *Srg3* CKO forelimb buds (S7B Fig; n = 4 per gene). These data suggest that the Srg3-containing complexes might be required to pattern proximal skeletons. As limb bud outgrowth distally proceeds, *Sox9*-expressing progenitors were also decreased in the zeugopod and autopod primordia of *Srg3* CKO forelimb buds (Fig 5C, middle and right panel; n = 8). Particularly, *Sox9*-expressing autopod progenitors in *Srg3* CKO forelimb buds did not initiate mesenchymal condensation. Furthermore, *Hoxa13*, which delineates the presumptive autopod territories [47], was distalized and relatively enhanced in the anterior region (Fig 5D; n = 5). Taken together, deficiency of mesenchymal *Srg3* progressively resulted in the loss of *Sox9*-positive progenitors in zeugopod and autopod primordia and this loss was paralleled by alterations in BMP activity.

### Genetic interaction between *Srg3* and *Twist1* reveals synergism in the anterior zeugopod development

We tested whether ectopic Shh activity impacts anterior zeugopod development, as demonstrated by the absence of the radius and hypoplastic tibia in *Srg3* CKO limbs. We introduced a single conditional allele of *Twist1* (*Twist1*^*f/+*^), which represses *Shh* expression in the anterior mesenchyme [41], into the *Srg3* CKO background. *Twist1*^*f/+*^;*Prx1Cre* forelimbs were phenotypically similar to *Srg3* CKO forelimbs, except for more severe defects in the scapula (n = 13/13) (compare S8A Fig with Fig 1B). However, *Twist1*^*f/+*^;*Srg3*^*f/f*^;*Prx1Cre* hindlimbs displayed ossification defects and syndactyly in the anterior autopods similar to those of *Srg3* CKO hindlimbs (Fig 1D and S8B Fig, arrow), but *Twist1*^*f/+*^;*Srg3*^*f/f*^;*Prx1Cre* hindlimbs exhibited a complete absence of tibia (S8B Fig, arrowhead). Consistent with this skeletal phenotype, *Sox9*-positive progenitors of the tibia primordia were reduced in *Twist1*^*f/+*^;*Srg3*^*f/f*^;*Prx1Cre* hindlimbs relative to *Twist1*^*f/+*^;*Prx1Cre* hindlimbs (S8C Fig; n = 6). In addition, ectopic expression of *Gli1* in *Twist1*^*f/+*^;*Srg3*^*f/f*^;*Prx1Cre* hindlimb buds was activated earlier than in *Twist1*^*f/+*^;*Prx1Cre* and *Srg3* CKO hindlimb buds (compare S8D Fig with S2B Fig; n = 7). However, early activation of ectopic *Shh* expression was not detected in *Twist1*^*f/+*^;*Srg3*^*f/f*^;*Prx1Cre* hindlimb buds (S8E Fig; n = 6). These data suggest that anterior zeugopod development might be affected by SWI/SNF complex-mediated epigenetic changes including the ectopic Hh pathway and not simply because of the Shh ligand-dependent pathway. This finding supports the idea that the fate of anterior skeletal progenitors is progressively determined.

### Bifunctional action of SWI/SNF complex in the Hh pathway regulates the spatiotemporal expression of *Grem1*

**Fig 6:**
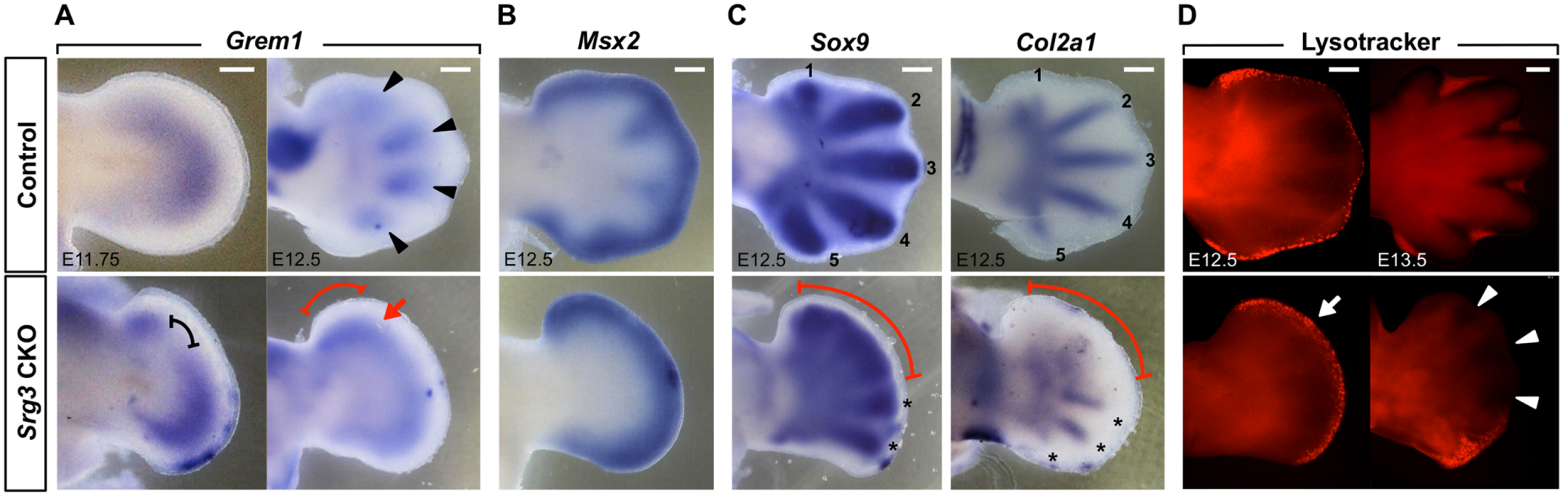
Bifunctional action of the SWI/SNF complex in the Hh pathway regulates the spatiotemporal expression of *Grem1*. **(A, B)** Distribution of *Grem1* and *Msx2* transcripts in control and *Srg3* CKO forelimb autopods at indicated stages. Black bracket points to the distance between two domains of *Grem1*. Spatial restriction to the interdigital mesenchyme (arrowheads), combined region of separate domains (arrow), and enhanced region (red bracket) of *Grem1* are indicated. **(C)** Expression pattern of *Sox9* and *Col2a1* in E12.5 control and *Srg3* CKO forelimb autopods. Brackets indicate the delayed region of chondrogenic differentiation. Numbers 1 to 5 indicate the primordia of digit rays 1 to 5. Asterisks indicate the preceding chondrogenesis of shortened digit primordia in the posterior. **(D)** Distribution of apoptotic cells by Lysotracker Red staining in control and *Srg3* CKO forelimb autopods at E12.5 and E13.5. Arrow points to increased cell death. Arrowheads indicate the reduction of interdigital cell death. Scale bars in (A−D): 200 μm.

The low to high transition of BMP activity by the timely termination of *Grem1* expression is required to initiate condensation and chondrogenic differentiation of proliferative digit progenitors [30, 43]. To determine the effect of bifunctional action of the SWI/SNF complex on chondrogenic differentiation, *Grem1* expression and BMP activity were analyzed at later stages. By E11.75, *Grem1* expression began to be downregulated throughout the entire mesenchyme of control forelimb buds, but its decline was not observed and its separated domains became closer than those at E11.5 in *Srg3* CKO forelimb buds (compare Fig 6A left panel with Fig 3C, black brackets; n = 8). At E12.5, *Grem1* expression was cleared from the presumptive digit territories and confined to the interdigital mesenchyme in control forelimbs, but these spatial pattern changes were not observed in the autopods of *Srg3* CKO forelimbs (Fig 6A, right panel; n = 6). Although *Msx2* expression in the anterior margin of *Srg3* CKO forelimb autopods was comparable to that in controls, it was undetectable in the interdigital mesenchyme (Fig 6B; n = 8). By contrast, the increased expression of *Grem1* and low BMP activity were observed in the anterior region of *Srg3* CKO hindlimb autopods (S9A Fig). We next examined whether delayed temporal kinetics of *Grem1* in *Srg3* CKO autopods is correlated with chondrogenesis of digit primordia and with digit separation. In *Srg3* CKO autopods, the distributions of *Sox9* and its target gene *Col2a1* revealed delayed mesenchymal condensations, and anterior digit progenitors were relatively less condensed than posterior ones (Fig 6C and S9B Fig; n = 8 per gene). In addition, the comparison of *Col2a1* distributions in *Srg3* CKO fore- and hindlimb autopods revealed that both the extent of *Grem1* propagation and its anterior upregulation caused the sequential onset of chondrogenesis in the posterior and anterior autopods. At this stage, Lysotracker Red staining in *Srg3* CKO forelimb autopods showed increases of apoptotic cells in the distal mesenchyme underlying the AER, likely as a consequence of growth defects (Fig 6D, left panel; n = 6 per stage). Furthermore, the reduction of cell death in the interdigital mesenchyme, resulting in soft tissue syndactyly, was observed in the anterior autopods of *Srg3* CKO forelimbs at E13.5 and hindlimbs at E12.5 (Fig 6D, right panel and S9C Fig). Taken together, these data indicate that the spatiotemporal regulation of *Grem1* by the SWI/SNF complex is involved in digit determinative processes as well as in cell survival of expanding autopod progenitors.

## Discussion

Our genetic analysis has shown that the SWI/SNF complex is required to modulate Shh responsiveness and repress the ectopic Hh pathway. Although specification of the AP limb bud axis is not affected by conditional inactivation of *Srg3* in the limb bud mesenchyme, *Srg3* CKO posterior progenitors fail to respond to graded Shh activity, leading to the redistribution of epithelial-mesenchymal signaling to the distal region. In parallel, loss of *Srg3* causes the activation of ligand-independent and subsequent ligand-dependent Hh pathway in the anterior mesenchyme, resulting in the loss of anterior identity over time. Our analysis also reveals the dual requirement of the SWI/SNF complex in the Hh pathway for spatiotemporal regulation of *Grem1*.

Posterior limb skeletal elements are patterned depending on Shh signaling [2, 4]. By contrast, recent reports have shown that formation of proximal and anterior limb skeletons is inhibited by early Hh activity prior to establishment of the ZPA and by activation of the anterior Hh pathway during limb patterning [10, 31]. Skeletal phenotypes in *Srg3* CKO forelimbs suggest that the Srg3-containing SWI/SNF complex is required for these distinct responses to Hh signaling. It has been known that SWI/SNF complexes and Polycomb group (PcG) proteins have antagonistic functions in repressing differentiation-related genes of embryonic stem cells [38]. In anterior limb buds, however, the SWI/SNF complexes appear to function synergistically with PcG proteins to repress the basal expression of Shh target genes. Consistent with our findings, deletion of H3K27 methyltransferase *Ezh2*, a catalytic subunit of PRC2, leads to ectopic expression of Shh target genes in anterior limb buds as well as derepression of Shh target genes in MEFs [39, 48]. Given that the PRC2 interacts with Gli proteins in developing limbs, PRC2 complexes are also likely to be involved in Gli-mediated repression of Shh target genes in anterior limb buds. In addition to the repressive function in the anterior limb bud, it is assumed that the SWI/SNF complexes also act cooperatively with H3K27 demethylases in activating Shh-induced target genes. It has been demonstrated that the SWI/SNF complexes functionally interact with H3K27 demethylases such as Jmjd3 and Utx in various tissues such as developing lungs and hearts [36, 37]. Particularly, a recent report showed changes in the epigenetic environment by switching Ezh2-PRC2 to Jmjd3 for Shh-induced target gene activation [39]. This implies that cooperative action between the SWI/SNF complex and Jmjd3 might be required for Shh target gene activation during limb development. Previous studies regarding SWI/SNF components have demonstrated that *Snf5* deficiency leads to ectopic expression of *Gli1* in developing limbs [49], and ATPase *Brg1* is involved in the regulation of Shh target genes in an ATPase activity-independent manner during neural development [50]. However, we have presented genetic evidence showing bifunctional action of the SWI/SNF complex in distinct territories of limb bud mesenchyme. We do not exclude the possibility that the SWI/SNF complex acts cooperatively with other chromatin regulators such as histone deacetylase (HDAC) that is associated with Shh/Gli pathway in developing limbs [50, 51]. In addition, the phenotypes observed in *Srg3* CKO limbs raise the possibility that the SWI/SNF complex likely controls the expression of other transcriptional regulators not specific to the Shh signaling pathway, such as Bmp and Hox genes. Further studies, including genome wide mapping of a H3K27Ac enhancer mark from the anterior and posterior limb buds of *Srg3* CKO embryos, will help to elucidate the distinct regulatory functions of the SWI/SNF complex in chondrogenic differentiation and proximal patterning.

In *Srg3* CKO forelimbs, one notable phenotype is the formation of variable digits, unlike polydactyly in hindlimbs. Concomitant deletion of Gli2 and Gli3 completely eliminates *Gli1* expression but does not lead to digit loss in developing limbs [4, 11, 18]. *Prx1Cre*-mediated early deletion of *Ptch1*, however, causes oligodactyly and is accompanied by activation of the Hh pathway, whereas late *Ptch1* depletion causes polydactyly [9, 10]. Importantly, we have uncovered the requirement of the SWI/SNF complex for robust expression of *Ptch1*. Thus, the core mesenchymal deficiency of *Ptch1* expression, resulting from its posterior restriction, may lead to reduced Shh activity sensing and restrain posterior digit formation in *Srg3* CKO forelimbs. In *Srg3* CKO forelimb buds, the reduced sensing of Shh causes distalization of epithelial-mesenchymal signaling and *Hoxa13*/*Hoxd13*-positive presumptive autopod regions, markedly similar to limb buds conditionally lacking *Ptch1* [9, 35]. Recent studies on the mammal species with two to four digits might support variable digit patterning by altered *Ptch1* expression observed in *Srg3* CKO forelimb buds [35, 52]. We assume that the extent of digit loss might be dependent on the integrity of the SWI/SNF complex controlled by Srg3. Meanwhile, ectopic *Shh* expression was induced in *Srg3* CKO limb buds, although there is no enrichment for Srg3 on the ZRS. It has been shown that ectopic expression of *Hoxd13* and *Hand2* leads to misexpression of *Shh* in anterior limb buds [53–55]. These molecular changes observed in *Srg3* CKO limb buds may result in ectopic expression of *Shh*, causing preaxial polydactyly. Taken together, variable digit patterning in *Srg3* CKO forelimbs appears to occur through combinatorial actions of altered *Ptch1* expression and ectopic anterior Hh activity.

Both the proximal and distal BMP activities in the anterior mesenchyme of *Srg3* CKO forelimb buds are distinct from those of *Gli3*-deficient limb buds [22]. The comparison of anterior zeugopod development and digit numbers between *Srg3* CKO fore- and hindlimbs showed that the dose and exposed duration of ectopic Hh activity negatively impact the differentiation of anterior prechondrogenic progenitors. Our data and previous reports have demonstrated that the expansion of Hh signaling has an inhibitory effect on the formation of proximal and anterior skeletal elements [10, 31, 41]. In this regard, the proliferative expansion of anterior progenitors negatively controlled by Gli3 might require time to ensure a sufficient population such as both *Irx3*- and *Irx5-*positive early progenitors [22, 31]. Particularly, the genetic interaction between *Srg3* and *Twist1* showed synergism in limb skeletal formation such as in anterior zeugopod development. *Twist1* not only functions as a Shh repressor but also controls the onset of osteoblast differentiation [41, 56]. It is possible that the repressive roles of *Twist1* in developmental processes might contribute to recruit chromatin regulators such as the PcG, for example, in promoting the epithelial-mesenchymal transition and in suppressing mesenchymal stem cell senescence [57, 58]. The functional interaction of the SWI/SNF complex with transcriptional regulators acting either as activators or as repressors, which can recruit enzymes that modify active or repressive histone marks, may reveal synergistic and antagonistic actions of gene regulation at the chromatin level.

Derepression is one of the regulatory mechanisms underlying limb bud patterning. Our data highlight the sustained requirement of the SWI/SNF complex for transcriptional regulation of *Grem1*, a major Gli target gene controlled by derepression [23]. The expression of *Grem1* in the limb bud is severely reduced in *Shh*^*−/−*^ mutants and symmetrically expanded in both *Gli3*^*−/−*^ and *Shh*^*−/−*^;*Gli3*^*−/−*^ mutants [16, 17, 59]. Compared with previous observations, *Grem1* expression in *Srg3* CKO forelimb buds is dynamically redistributed, possibly a consequence of the reconstitution of the GliA/GliR gradient by low Shh responsiveness and ectopic Shh activity. Consistently, it has recently been suggested that limb-specific enhancers integrated by multiple posterior GliA- and anterior GliR-dependent CRMs regulate the transcriptional activity of *Grem1* [60]. Furthermore, the combined region of *Grem1* expression domains in *Srg3* CKO forelimb buds indicates that the definitive digit identity in this region could be progressively determined by altered Hh activity (Fig 6). Thus, our analysis suggests that bifunctional action of the SWI/SNF complex in the Hh pathway is essential for spatiotemporal regulation of *Grem1* that mediates AP skeletal patterning elicited by GliA and GliR functions [18, 22].

We have demonstrated that the SWI/SNF complex plays decisive roles in conferring graded Shh signaling upon developing limb progenitor cells. The SWI/SNF complex influences the progression of interlinked morphogen signaling pathways by modulating Shh responsiveness in the posterior limb bud and by repressing the Hh pathway in Shh-free regions. Our study showing the effects of epigenetic regulation by the SWI/SNF chromatin remodeling complex on limb patterning provides insights into deciphering developmental processes directed by morphogen gradients.

## Materials and Methods

### Ethics statement

All experiments with animals were performed according to the guidelines established by the Seoul National University Institutional Animal Care and Use Committees (SNUIACUC). SNUIACUC approved this study (approval number: SNU-130503-2). CO_2_ gas was used for animal euthanasia.

### Mice and embryos

Generation of mice carrying a conditional allele of *Srg3* (*Srg3*^*f/f*^) was previously described [28]. *Srg3*^*f/f*^, *Prx1Cre* [29], and *Twist1*^*f/f*^ mice [41] were bred and maintained on a C57BL/6J genetic background. For all experiments, *Srg3*^*+/+*^;*Prx1Cre* and *Srg3*^*f/+*^;*Prx1Cre* mice and embryos harboring a *Prx1Cre* transgene were used as wild-type controls.

### Whole-mount *in situ* hybridization

The transcript distributions were assessed by whole-mount *in situ* hybridization according to the standard procedures as described [61] with the following minor modifications: embryos were permeabilized in proteinase K (10 μg/ml) in PBST at room temperature for 11 min (E9.5−E10.5), 14 min (E10.5−E11.5) or 17 min (E11.5−E12.5) for analysis of limb mesenchyme and briefly for 3 min regardless of age for analysis of AER. All probes were linearized with the appropriate restriction enzyme and labeled using digoxigenin RNA labeling mix (Roche) with the appropriate polymerase (T7, T3 or SP6). *Shh*, *Gli1*, *Bmp2*, *Bmp4* and *Bmp7* probes were kindly provided by Y. Kong (Seoul National University). *Fgf4* (Addgene plasmid #22085) [62] and *Fgf8* (Addgene plasmid #22088) [63] probes were gifts from G. Martin. *Hoxa9*, *Hoxd9* and *Hoxd10* probes were generously provided by D. Wellik and *Irx3* and *Irx5* probes were provided by C. Hui. Other probes were amplified by PCR from cDNA fragments encompassing at least two exons (about 400−600 bp) of target genes and cloned into pGEM-T vectors (Promega). All representative expression patterns were obtained by analyzing at least three independent embryos per probe.

### Skeletal staining and detection of apoptotic cells

Skeletal preparations and detection of apoptotic cells were performed as previously described [19, 30]. For analysis of skeletal structures, samples were collected at E14.5 and P0 and cartilages and bones were stained with Alcian Blue and Alizarin Red, respectively. Distribution of apoptotic cells in whole limb buds was analyzed using Lysotracker Red (Molecular Probes L-7528, Invitrogen).

### Cell culture

Primary Mouse Embryonic Fibroblasts (MEFs) prepared from E13.5 *Srg3*^*f/f*^ embryos, HEK293T, and Phoenix-eco cells were grown in DMEM medium (WelGENE) supplemented with 10% fetal bovine serum (FBS). For generation of *Srg3*-deficient MEFs, Phoenix-eco packaging cells were transfected with retroviral vectors expressing GFP alone (Empty) as a control or Cre-recombinase (Cre) by calcium phosphate method and their retroviral supernatants were harvested 2 d after transfection. MEFs were infected with the retroviral supernatant by spin infection for 90 min at 2500 rpm in the presence of 8 μg/ml polybrene. For inhibition of Hh signaling, MEFs were treated with 5 μM cyclopamine dissolved in ethanol vehicle for 24 h. For Shh stimulation, HEK293T cells were transiently transfected with ShhN expressing vector (kindly provided by M. Kang, Korea University Guro Hospital). Shh conditioned medium produced from transfected HEK293T cells was replaced with DMEM containing 2% FBS 24 h before harvesting and filtering of medium, and then added to MEFs for 24 h. Shh stimulated or cyclopamine treated MEFs were harvested for qPCR.

### Immunoprecipitation (IP) and western blotting

IP and western blotting were performed as previously described [19, 28]. Limb bud lysates were immunoprecipitated or detected with following antibodies: Gli2 (R&D systems), Gli3 (R&D systems), α-tubulin (Sigma), Ezh2 (BD transduction), Suz12 (Cell signaling), H3K27me3 (Millipore), Histone H3 (Abcam), and rabbit polyclonal IgG (Millipore). Antisera for Brg1 and Srg3 were raised from rabbits in our laboratory. The band density of Gli3R level was quantified using ImageJ software (NIH) and normalized to α-tubulin as a loading control.

### Chromatin immunoprecipitation (ChIP)

E11.5 control and *Srg3*^*f/f*^;*Prx1Cre* limb buds were dissected in cold PBS and minced with a douncer and MEFs were trypsinized. Dissociated tissues and MEFs were crosslinked in 1% formaldehyde (Sigma) for 10 min on a rotator at RT and were lysed for 10 min on ice with SDS lysis buffer (1% SDS, 50mM Tris-Cl (pH 8.1), 10mM EDTA). Lysates were sonicated to an average length of 200–500 bp using a Bioruptor sonicator and diluted 10-folds in dilution buffer (20mM Tris-Cl (pH 8.1), 150mM NaCl, 1% Triton X-100, 2mM EDTA). To reduce nonspecific background, samples were precleared for minimally 1 h with salmon-sperm DNA/Protein-A or G agarose (50% slurry, Millipore). Precleared lysates were incubated overnight on a rotator at 4°C with anti-Brg1, anti-Srg3, anti-H3K27me3 (Millipore), anti-Gli2 (abcam), anti-Gli3 (R&D systems) or with isotype-control anti-rabbit IgG (Millipore) as a negative control. Washing, elution and reverse-crosslinking of DNA-immunocomplexes and DNA purification were enriched as previously described [28]. Purified DNA was analyzed by qPCR with the following primers:

g1 forward: 5’-CCGGCACCCCCTCTCTAG-3’,

g1 reverse: 5’- GGCTCTTCCCGCTCACTTC-3’,

g2 forward: 5’-TTGCTCCCCGCTCTGAATC-3’,

g2 reverse: 5’-CTTGATGCTGTTCCCAAAGCT-3’,

p1 forward: 5’-AGGACACAATGCACCTGAGG-3’

p1 reverse: 5’-AGGTCTTGTGGGTGCCTCTA-3’

p2 forward: 5’-TAGTGGCGAGAATGACAGCG-3’

p2 reverse: 5’-TTTCTCCCTACCAACCGCAG-3’,

p3 forward: 5’-ACACACTGGCGCACTATCCA-3’,

p3 reverse: 5’-CCTCAAGCTGCAGCAAATACTG-3’,

p4 forward: 5’-GAATGGGAGAGGGAGGAAAGAT-3’,

p4 reverse: 5’-GCGGGAGCTCAGTTAGGAAA-3’,

p5 forward: 5’-TCTTCCAGCATGCTTACCTCTTT-3’,

p5 reverse: 5’-GCTTGGCCGCTGTAATCAAA-3’.

## Supporting Information

S1 Fig *Prx1Cre*-mediated inactivation of *Srg3* in the limb bud mesenchyme and skeletal phenotypes of *Srg3* CKO limbs. **(A)** Whole-mount *in situ* hybridization reveals the distribution of *Srg3* transcripts in E9.5 forelimb buds and E10.5 hindlimb buds of control and *Srg3* CKO embryos. Scale bars: 100 μm. **(B)** Immunoblot analysis of Brg1 and Srg3 proteins in E10.5 control and *Srg3* CKO forelimb buds and hindlimb buds. α-tubulin was used as loading control. **(C)** Skeletal structures of zeugopod elements in hindlimbs of control and *Srg3* CKO pups (P0) and embryos (E14.5). Tibia (*ti*) and fibula (*fi*) were shortened in *Srg3* CKO hindlimbs compared with control. **(D)** Bright-field images of control and *Srg3* CKO autopods at E16. Arrows indicate syndactyly in the anterior region of *Srg3*-deficient autopods.
(TIFF)

S2 Fig Formation of limb signaling centers and anterior ectopic activation of Hh pathway in *Srg3* CKO limb buds. **(A)** The expression of *Shh* and *Fgf8* sensing the ZPA and the AER, respectively, in control and *Srg3* CKO forelimb buds at E10. Arrowhead indicates the reduced activity of AER. **(B)** The distribution of *Gli1* and *Ptch1* transcript in control and *Srg3* CKO hindlimb buds at indicated stages. Anterior ectopic expression of *Gli1* in *Srg3* CKO hindlimb buds was observed later than that in mutant forelimb buds. Arrowheads indicate anterior ectopic expression. Scale bars in (A−B): 100 μm.
(TIFF)

S3 Fig The extent of loss of anterior identity in *Srg3* CKO limb buds correlates with the timing of *Srg3* inactivation. **(A, B)** The expression of anterior marker genes *Alx4* and *Pax9* in E11.5 control and *Srg3* CKO forelimb buds (A) and hindlimb buds (B) is indicated. Scale bars: 100 μm.
(TIFF)

S4 Fig Expression pattern of epithelial-mesenchymal signaling genes in control and *Srg3* CKO hindlimb buds at late stages and the enrichment of Srg3 on the ZRS. **(A**, **C−E)** Spatial distribution of *Shh*, *Grem1*, *Fgf8* and *Hoxd13* in control and *Srg3* CKO hindlimb buds. Red arrowheads denote anterior ectopic expression. The anterior expansion of *Shh* (A, arrow) and the distalized expression of *Grem1* (C, red brackets) and *Hoxd13* (E, asterisk) were observed in *Srg3* CKO hindlimb buds, similarly to forelimb buds. Scale bars: 100 μm. (**B**) ChIP−qPCR analyses of DNA fragments precipitated with anti-Srg3 and IgG in E11.5 control limb buds. Bottom panel is a schematic representation of the relative positions of primer sets (blue lines: z1-z10) using the sequence of ZRS region within intron 5 of *Lmbr1* gene. Gli binding region (g1) was used as a positive DNA control and anti-IgG as a negative antibody control.
(TIFF)

S5 Fig Loss of *Srg3* does not lead to global change in the level of H3K27me3 in MEFs and in the anterior and posterior limb buds. (**A**) Gli2 and Gli3 proteins interact with PRC2 components Ezh2 and Suz12 in developing limbs. PC indicates the preclear beads as a negative control. (**B**, **C**) Immunoblot analysis of H3K27me3 in *Srg3*-deficient MEFs (**B**) and in the anterior and posterior regions of *Srg3* CKO forelimb buds (**C**). Histone H3 was used as a loading control.
(TIFF)

S6 Fig Expression pattern of *Bmp2* and *Bmp7* in E11.75 control and *Srg3* CKO forelimb buds. Arrowheads indicate the posterior domain of *Bmp2* transcript. Scale bars: 200 μm.
(TIFF)

S7 Fig Expression pattern of proximal skeletal patterning-related genes in control and *Srg3* CKO forelimb buds. (**A**) The expression of *Hoxa9*, *Hoxd9*, *Hoxd10* in control and *Srg3* CKO hindlimb buds at indicated stages. (**B**) The expression of *Irx3* and *Irx5* in control and *Srg3* CKO hindlimb buds at indicated stages. Scale bars in (A−B): 200 μm.
(TIFF)

S8 Fig Genetic interaction between *Srg3* and *Twist1* reveals the synergistic effect in the anterior zeugopod development. (**A**) Skeletal preparations from *Twist1*^*f/+*^;*Prx1Cre* and *Twist1*^*f/+*^;*Srg3*^*f/f*^;*Prx1Cre* forelimbs at P0. Red arrowhead indicates the defect in scapula development. **(B)** Skeletal preparations from *Twist1*^*f/+*^;*Prx1Cre* and *Twist1*^*f/+*^;*Srg3*^*f/f*^;*Prx1Cre* hindlimbs at P0. Red arrowhead indicates loss of tibia. Red arrow indicates the ossification defects and syndactyly in anterior autopod. fe, femur; fi, fibula; pg, pelvic girdle; ti, tibia. Scale bars in (A−B): 1mm. **(C−E)** Expression pattern of *Sox9*, *Gli1* and *Shh* in *Twist1*^*f/+*^;*Prx1Cre* and *Twist1*^*f/+*^;*Srg3*^*f/f*^;*Prx1Cre* hindlimb buds at indicated stages. Red arrowhead indicates the reduction of *Sox9* expression in the tibia primordia. Scale bars: 100 μm
(TIFF)

S9 Fig The increase of *Grem1* expression in the anterior mesenchyme induces the sequential onset of chondrogenesis in the posterior and anterior autopods of *Srg3* CKO hindlimbs. **(A, B)** Distribution of *Grem1*, *Msx2*, *Sox9* and *Col2a1* transcripts in control and *Srg3* CKO hindlimbs at indicated stages. Red brackets in (A) indicate the upregulated region of *Grem1* expression and the downregulated region of *Msx2* expression in *Srg3* CKO hindlimb autopods. Arrowheads in (B) mark the initiation of mesenchymal condensation giving rise to digit ray primordia. Red brackets denote the region of delayed chondrogenesis. Numbers 1 to 5 indicate the primordia of digits 1 to 5. Asterisk indicates reduced digit primordia. **(C)** Lysotracker Red staining reveals the decrease of apoptotic cells in the anterior interdigital mesenchyme of *Srg3* CKO hindlimb autopod (arrowheads). Scale bars in (A−C): 100 μm
(TIFF)
