# Supplementary figures and images for: Anteroposterior Limb Skeletal Patterning Requires the Bifunctional Action of SWI/SNF Chromatin Remodeling Complex in Hedgehog Pathway
Source: PLoS Genet. 2016 Mar 9;12(3):e1005915. doi: 10.1371/journal.pgen.1005915 (PMC4784730; doi:10.1371/journal.pgen.1005915)

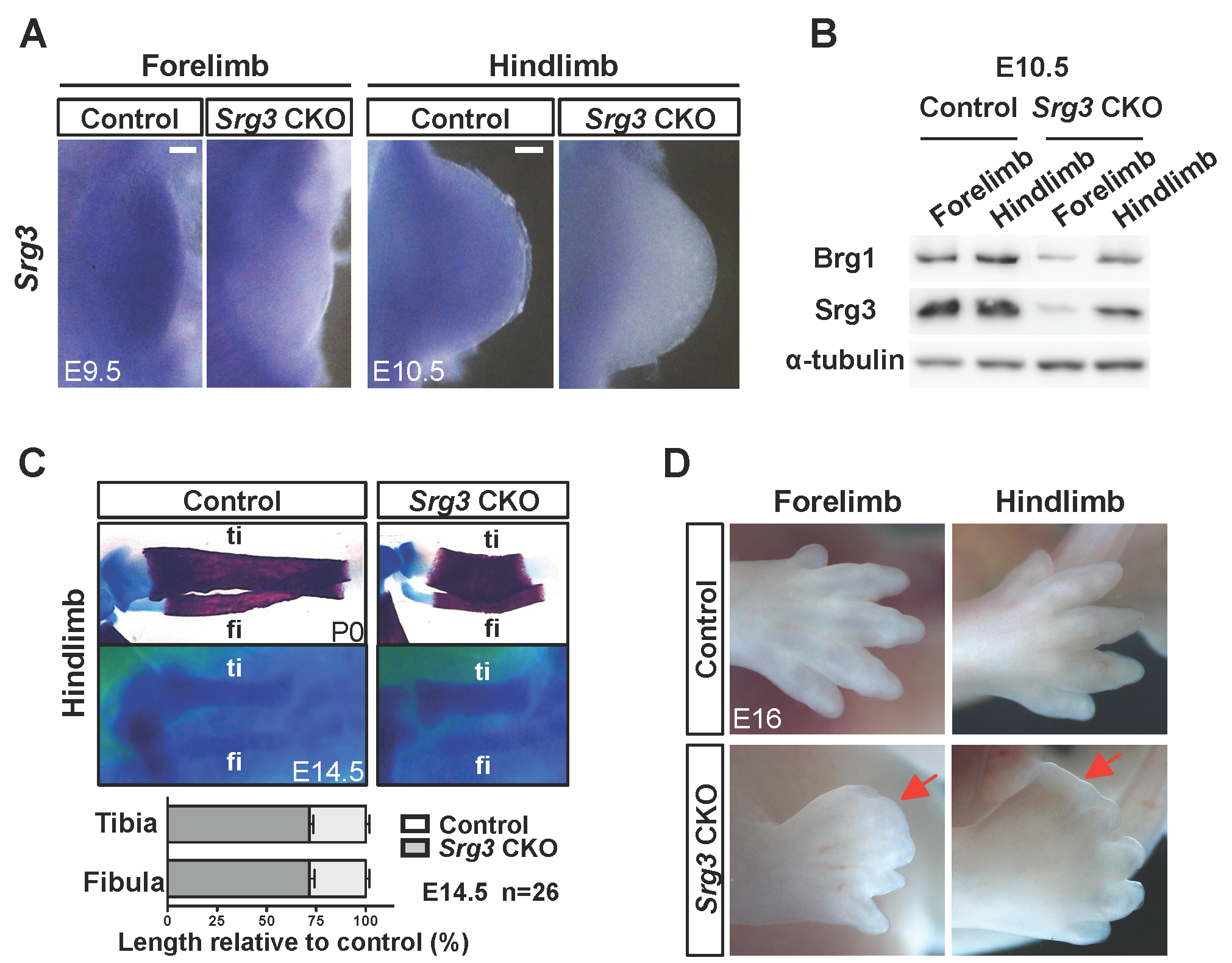

Supplement: S1 Fig — (A) Whole-mount in situ hybridization reveals the distribution of Srg3 transcripts in E9.5 forelimb buds and E10.5 hindlimb buds of control and Srg3 CKO embryos. Scale bars: 100 μm. (B) Immunoblot analysis of Brg1 and Srg3 proteins in E10.5 control and Srg3 CKO forelimb buds and hindlimb buds. α-tubulin was used as loading control. (C) Skeletal structures of zeugopod elements in hindlimbs of control and Srg3 CKO pups (P0) and embryos (E14.5). Tibia (ti) and fibula (fi) were shortened in Srg3 CKO hindlimbs compared with control. (D) Bright-field images of control and Srg3 CKO autopods at E16. Arrows indicate syndactyly in the anterior region of Srg3-deficient autopods. (TIFF) [file pgen.1005915.s001.tiff]

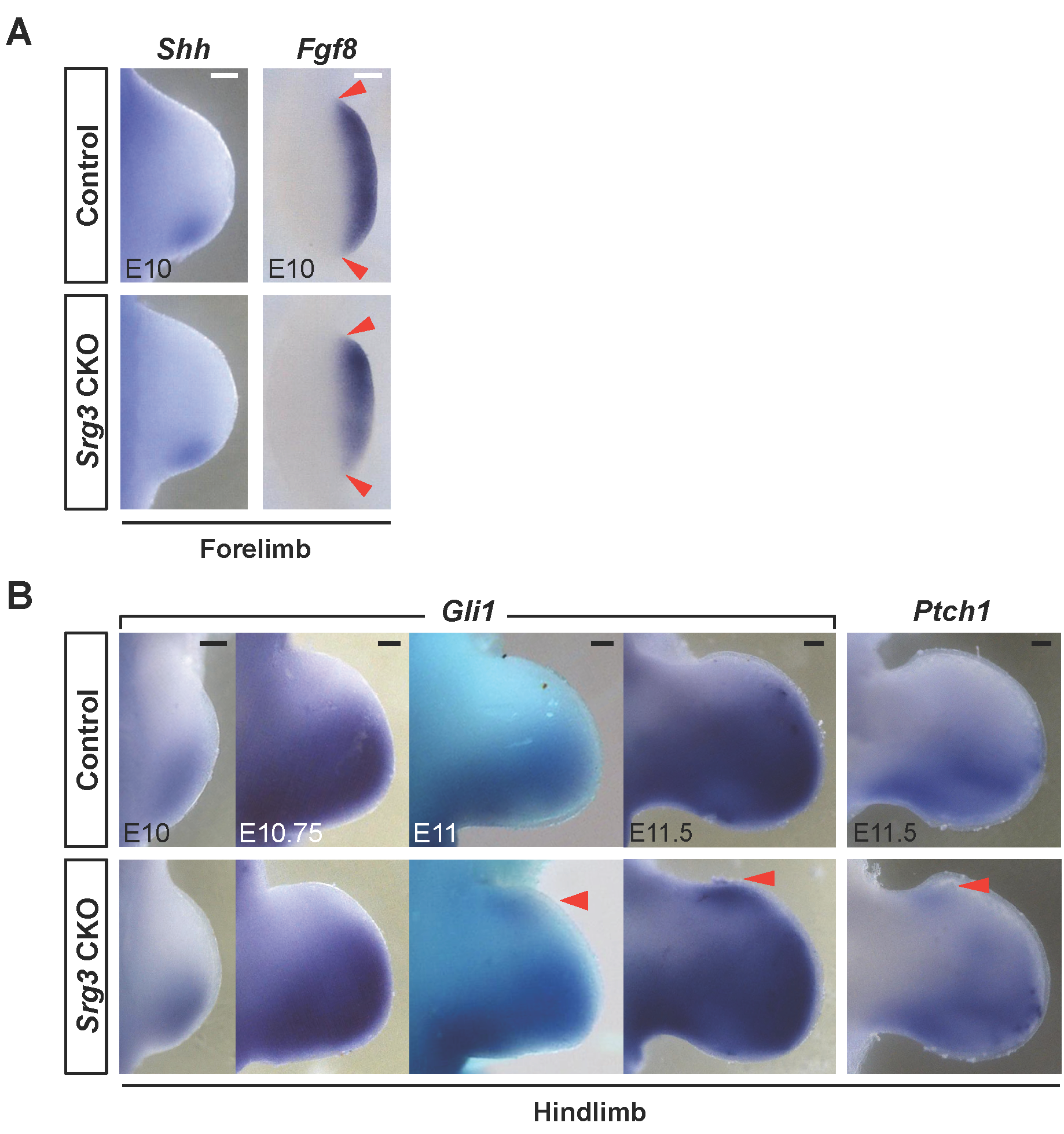

Supplement: S2 Fig — (A) The expression of Shh and Fgf8 sensing the ZPA and the AER, respectively, in control and Srg3 CKO forelimb buds at E10. Arrowhead indicates the reduced activity of AER. (B) The distribution of Gli1 and Ptch1 transcript in control and Srg3 CKO hindlimb buds at indicated stages. Anterior ectopic expression of Gli1 in Srg3 CKO hindlimb buds was observed later than that in mutant forelimb buds. Arrowheads indicate anterior ectopic expression. Scale bars in (A−B): 100 μm. (TIFF) [file pgen.1005915.s002.tiff]

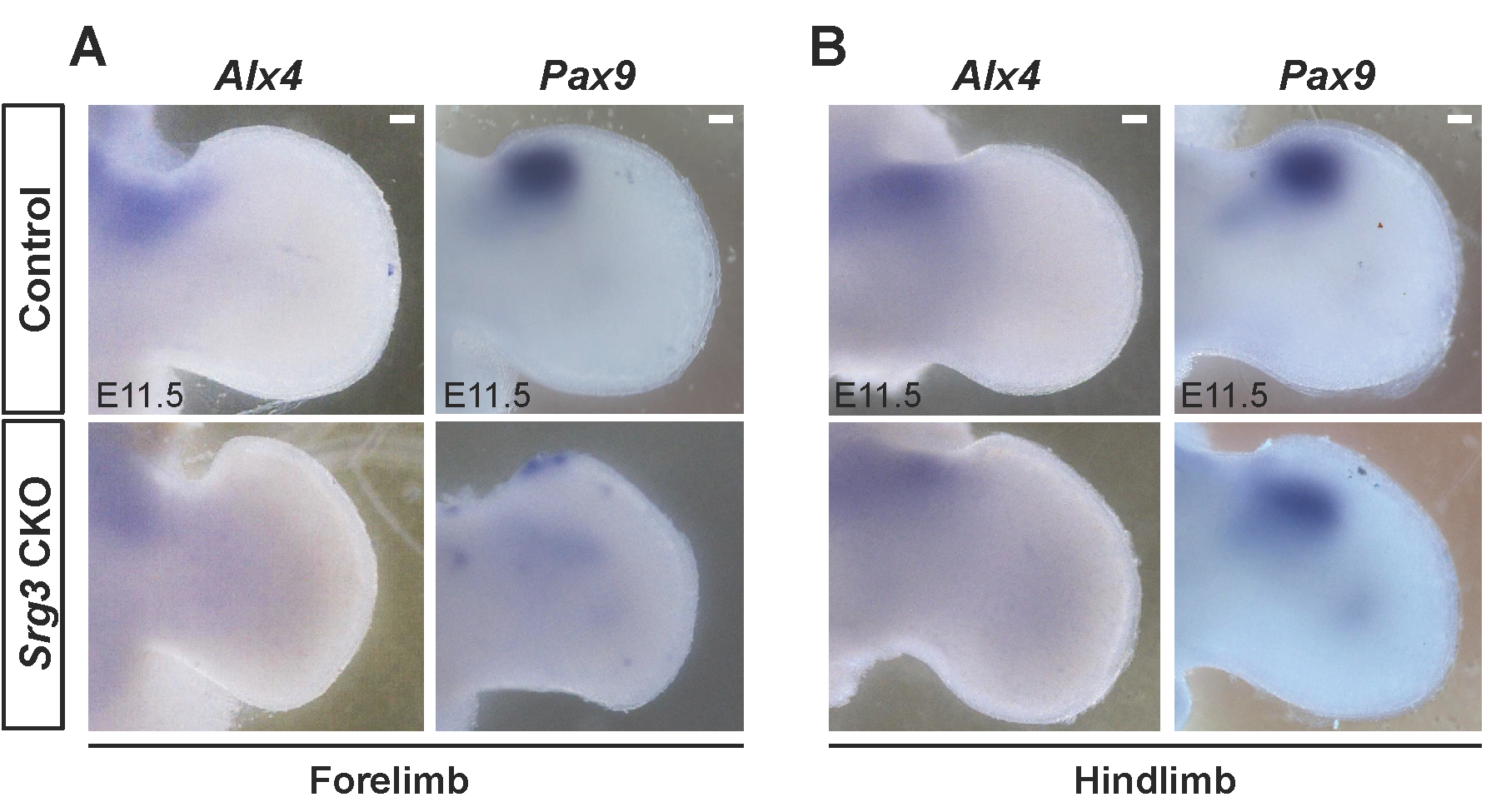

Supplement: S3 Fig — (A, B) The expression of anterior marker genes Alx4 and Pax9 in E11.5 control and Srg3 CKO forelimb buds (A) and hindlimb buds (B) is indicated. Scale bars: 100 μm. (TIFF) [file pgen.1005915.s003.tiff]

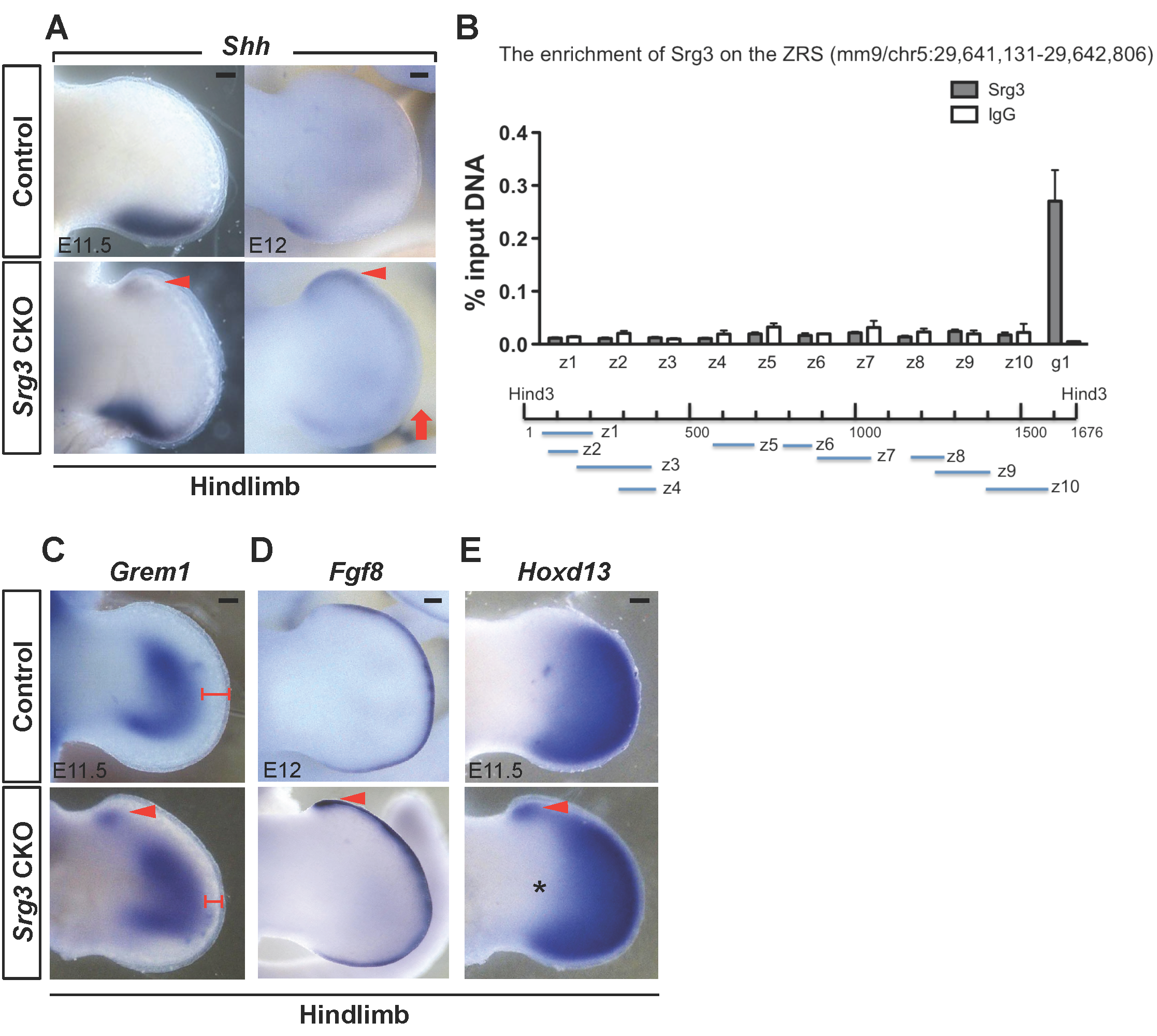

Supplement: S4 Fig — (A, C−E) Spatial distribution of Shh, Grem1, Fgf8 and Hoxd13 in control and Srg3 CKO hindlimb buds. Red arrowheads denote anterior ectopic expression. The anterior expansion of Shh (A, arrow) and the distalized expression of Grem1 (C, red brackets) and Hoxd13 (E, asterisk) were observed in Srg3 CKO hindlimb buds, similarly to forelimb buds. Scale bars: 100 μm. (B) ChIP−qPCR analyses of DNA fragments precipitated with anti-Srg3 and IgG in E11.5 control limb buds. Bottom panel is a schematic representation of the relative positions of primer sets (blue lines: z1-z10) using the sequence of ZRS region within intron 5 of Lmbr1 gene. Gli binding region (g1) was used as a positive DNA control and anti-IgG as a negative antibody control. (TIFF) [file pgen.1005915.s004.tiff]

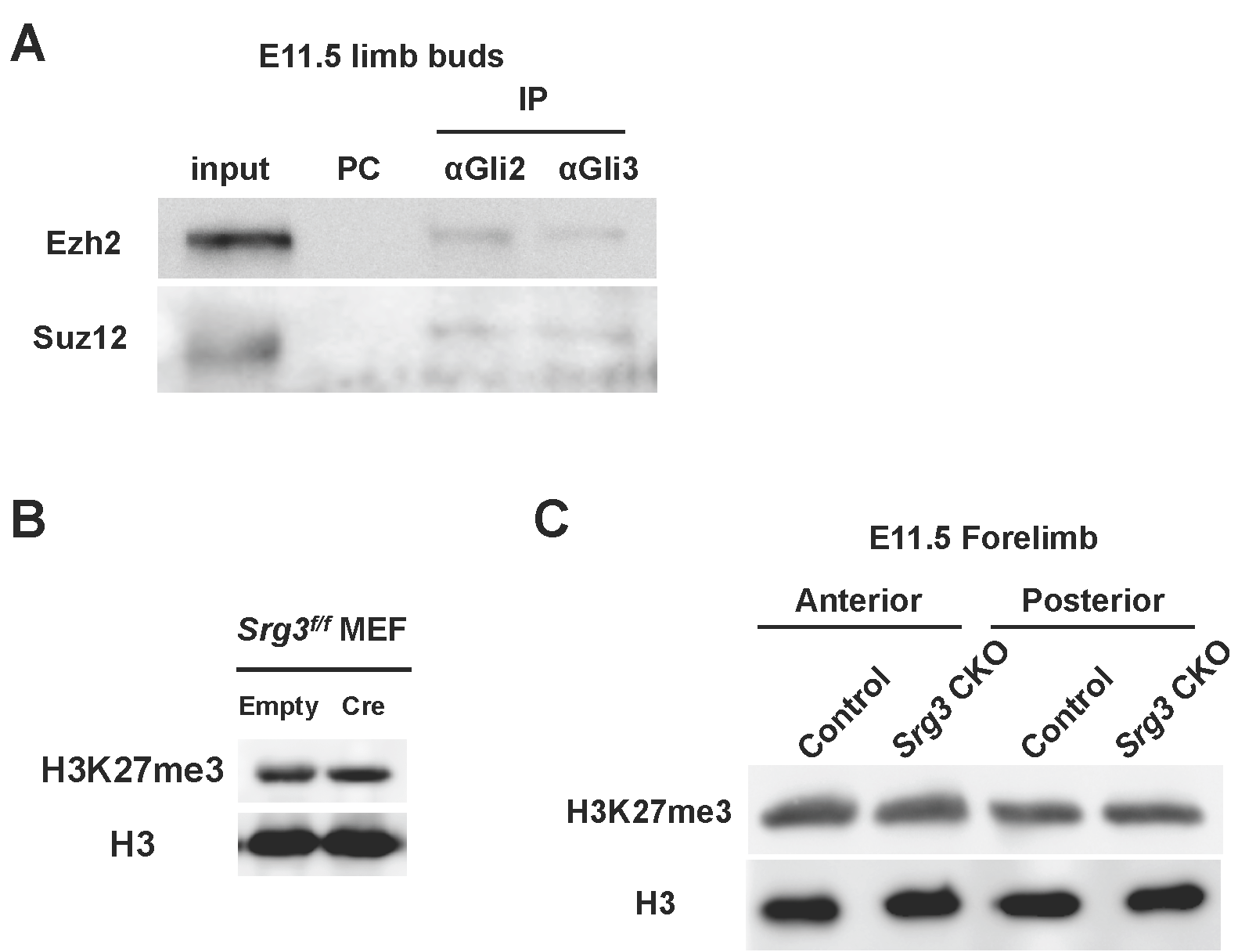

Supplement: S5 Fig — (A) Gli2 and Gli3 proteins interact with PRC2 components Ezh2 and Suz12 in developing limbs. PC indicates the preclear beads as a negative control. (B, C) Immunoblot analysis of H3K27me3 in Srg3-deficient MEFs (B) and in the anterior and posterior regions of Srg3 CKO forelimb buds (C). Histone H3 was used as a loading control. (TIFF) [file pgen.1005915.s005.tiff]

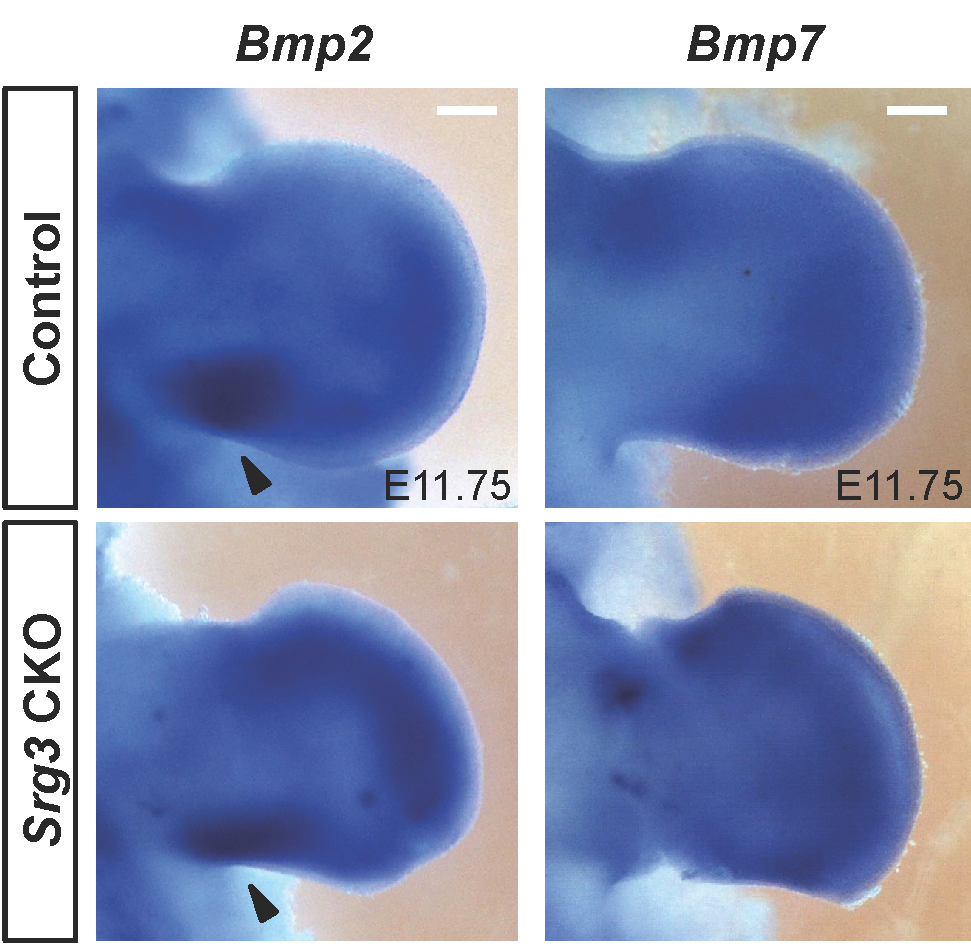

Supplement: S6 Fig — Arrowheads indicate the posterior domain of Bmp2 transcript. Scale bars: 200 μm. (TIFF) [file pgen.1005915.s006.tiff]

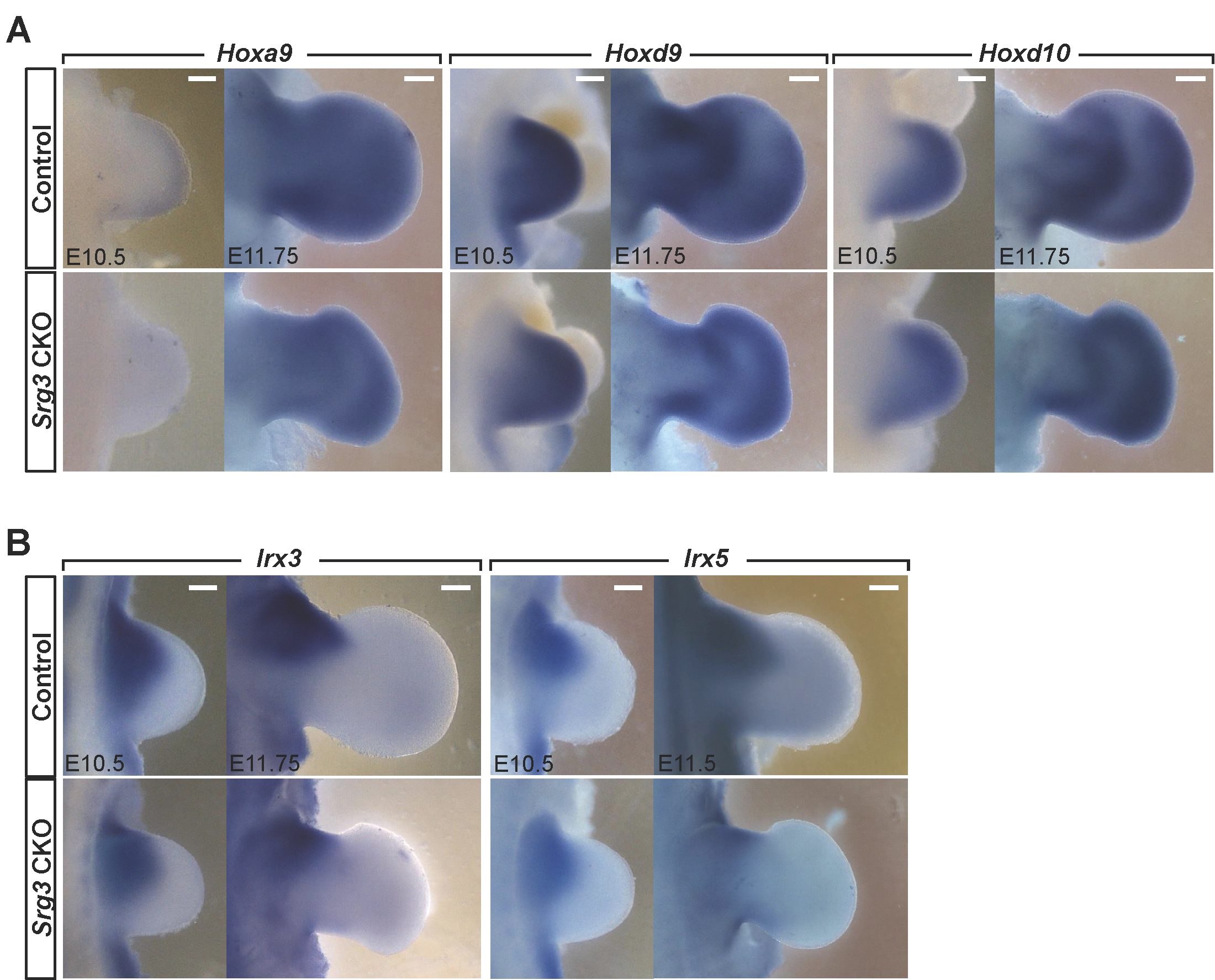

Supplement: S7 Fig — (A) The expression of Hoxa9, Hoxd9, Hoxd10 in control and Srg3 CKO hindlimb buds at indicated stages. (B) The expression of Irx3 and Irx5 in control and Srg3 CKO hindlimb buds at indicated stages. Scale bars in (A−B): 200 μm. (TIFF) [file pgen.1005915.s007.tiff]

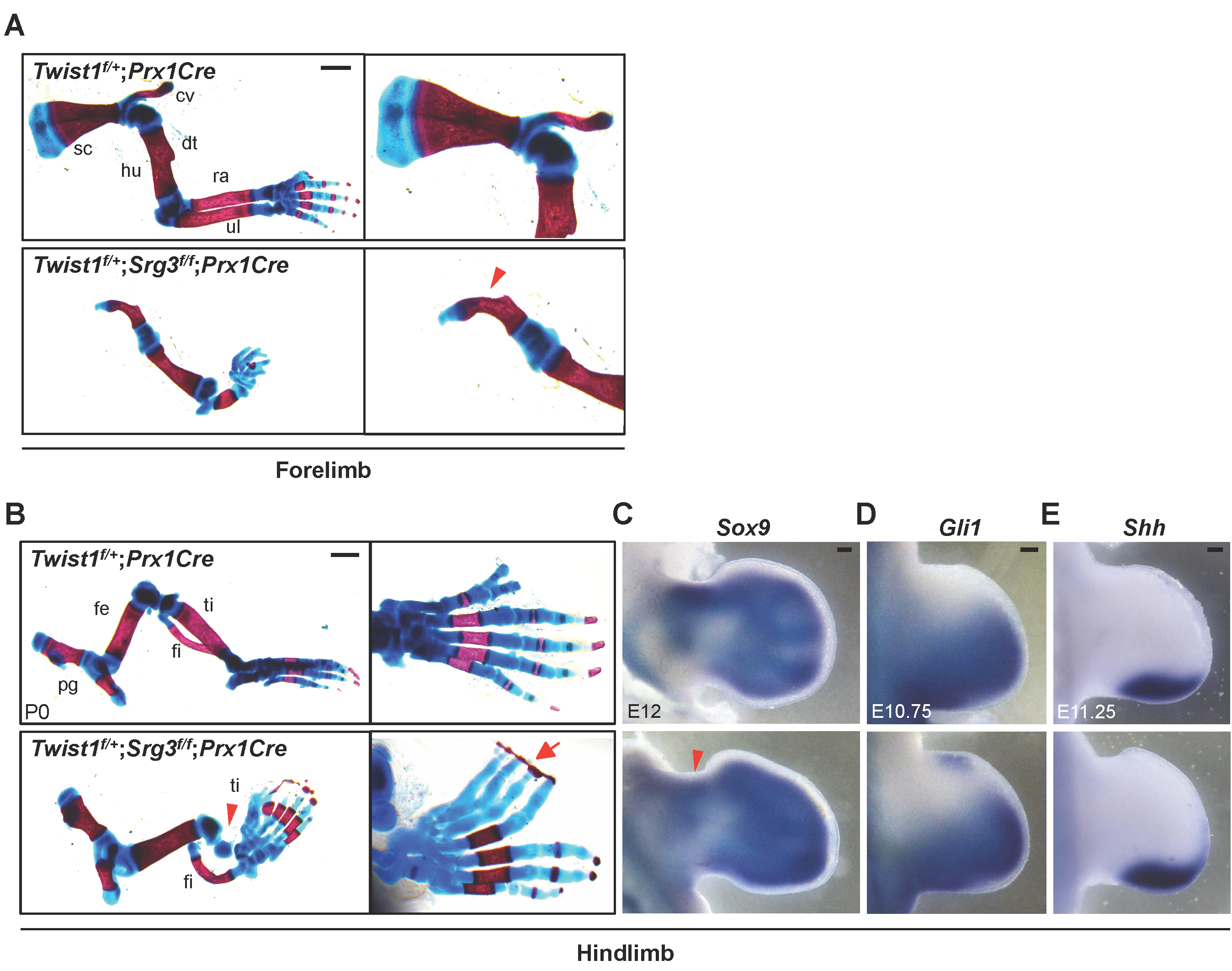

Supplement: S8 Fig — (A) Skeletal preparations from Twist1f/+;Prx1Cre and Twist1f/+;Srg3f/f;Prx1Cre forelimbs at P0. Red arrowhead indicates the defect in scapula development. (B) Skeletal preparations from Twist1f/+;Prx1Cre and Twist1f/+;Srg3f/f;Prx1Cre hindlimbs at P0. Red arrowhead indicates loss of tibia. Red arrow indicates the ossification defects and syndactyly in anterior autopod. fe, femur; fi, fibula; pg, pelvic girdle; ti, tibia. Scale bars in (A−B): 1mm. (C−E) Expression pattern of Sox9, Gli1 and Shh in Twist1f/+;Prx1Cre and Twist1f/+;Srg3f/f;Prx1Cre hindlimb buds at indicated stages. Red arrowhead indicates the reduction of Sox9 expression in the tibia primordia. Scale bars: 100 μm (TIFF) [file pgen.1005915.s008.tiff]

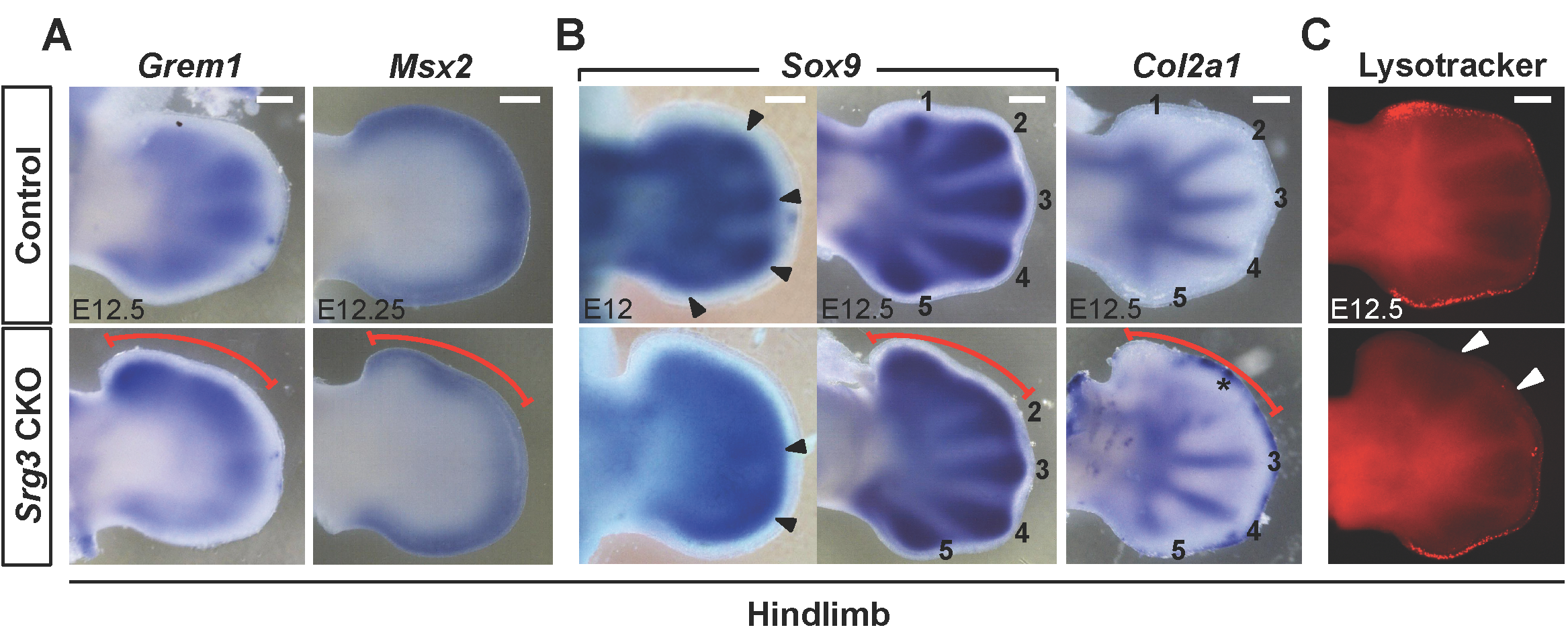

Supplement: S9 Fig — (A, B) Distribution of Grem1, Msx2, Sox9 and Col2a1 transcripts in control and Srg3 CKO hindlimbs at indicated stages. Red brackets in (A) indicate the upregulated region of Grem1 expression and the downregulated region of Msx2 expression in Srg3 CKO hindlimb autopods. Arrowheads in (B) mark the initiation of mesenchymal condensation giving rise to digit ray primordia. Red brackets denote the region of delayed chondrogenesis. Numbers 1 to 5 indicate the primordia of digits 1 to 5. Asterisk indicates reduced digit primordia. (C) Lysotracker Red staining reveals the decrease of apoptotic cells in the anterior interdigital mesenchyme of Srg3 CKO hindlimb autopod (arrowheads). Scale bars in (A−C): 100 μm (TIFF) [file pgen.1005915.s009.tiff]
